# Uncovering new *Firmicutes* species in vertebrate hosts through metagenome-assembled genomes with potential for sporulation

**DOI:** 10.1128/spectrum.02113-24

**Published:** 2024-09-16

**Authors:** Douglas Terra Machado, Beatriz do Carmo Dias, Rodrigo Cayô, Ana Cristina Gales, Fabíola Marques de Carvalho, Ana Tereza Ribeiro Vasconcelos

**Affiliations:** 1 Laboratório de Bioinformática, Laboratório Nacional de Computação Científica, Quitandinha, Petrópolis, Rio de Janeiro, Brazil; 2 Laboratory of Environmental Antimicrobial Resistance (LEARN), Departamento de Ciências Biológicas (DCB), Instituto de Ciências Ambientais, Químicas e Farmacêuticas (ICAQF), Universidade Federal de São Paulo (UNIFESP), Unidade José Alencar, Centro, Diadema, São Paulo, Brazil; 3 Laboratório ALERTA, Division of Infectious Diseases, Escola Paulista de Medicina (EPM), Universidade Federal de São Paulo (UNIFESP), São Paulo, Brazil; Lerner Research Institute, Cleveland, Ohio, USA

**Keywords:** metagenome-assembled genomes, metagenomics, bacterial sporulation, microbiome, *Firmicutes*

## Abstract

Metagenome-assembled genomes (MAGs) have contributed to identifying non-culturable microorganisms and understanding their ecological functions. MAGs offer an advantage in investigating sporulation-associated genes, especially given the difficulty of isolating many species residing in the gut microbiota of multiple hosts. Bacterial sporulation is a key survival mechanism with implications for pathogenicity and biotechnology. Here, we investigate MAGs from vertebrate hosts, emphasizing taxonomic identification and identifying sporulation-associated genes in potential novel species within the *Firmicutes* phylum. We identified potential new species in the classes *Clostridia* (*Borkfalkiaceae*, *Lachnospiraceae*, *Monoglobaceae*, and *Oscillospiraceae* families) and *Bacilli* (*Bacillaceae* and *Erysipelotrichaceae* families) through phylogenetic and functional pathway analyses, highlighting their sporulation potential. Our study covers 146 MAGs, 124 of them without refined taxonomic assignments at the family level. We found that *Clostridia* and *Bacilli* have unique sporulation gene profiles in the refined family MAGs for cattle, swine, poultry, and human hosts. The presence of genes related to Spo0A regulon, engulfment, and spore cortex in MAGs underscores fundamental mechanisms in sporulation processes in currently uncharacterized species with sporulation potential from metagenomic dark matter. Furthermore, genomic analyses predict sporulation potential based on gene presence, genome size, and metabolic pathways involved in spore formation. We emphasize MAGs covering families not yet characterized through the phylogenetic analysis, and with extensive potential for spore-forming bacteria within *Clostridia*, *Bacilli*, UBA4882, and UBA994 classes. These findings contribute to exploring spore-forming bacteria, which provides evidence for novel species diversity in multiple hosts, their adaptive strategies, and potential applications in biotechnology and host health.

**IMPORTANCE:**

Spores are essential for bacterial survival in harsh environments, facilitating their persistence and adaptation. Exploring sporulation-associated genes in metagenome-assembled genomes (MAGs) from different hosts contributes to clinical and biotechnological domains. Our study investigated the extent of genes associated with bacterial sporulation in MAGs from poultry, swine, cattle, and humans, revealing these genes in uncultivated bacteria. We identified potential novel *Firmicutes* species with sporulation capabilities through phylogenetic and functional analyses. Notably, MAGs belonging to *Clostridia*, *Bacilli*, and unknown classes, namely UBA4882 and UBA994, remained uncharacterized at the family level, which raises the hypothesis that sporulation would also be present in these genomes. These findings contribute to our understanding of microbial adaptation and have implications for microbial ecology, underlining the importance of sporulation in *Firmicutes* across different hosts. Further studies into novel species and their sporulation capability can contribute to bacterial maintenance mechanisms in various organisms and their applications in biotechnology studies.

## INTRODUCTION

The advent of culture-independent methods has expanded our comprehension of microbial diversity, enabling the exploration of microbial communities in environments where traditional cultivation methods would not be effective (1, 2). Among these approaches, shotgun metagenomics has emerged as a powerful tool, allowing for the reconstruction of metagenome-assembled genomes (MAGs) and characterizing microbial communities directly from environmental samples (3–5). By detecting both cultivable and non-cultivable microorganisms and their associated genes, metagenomic analysis offers a comprehensive understanding of microbial ecology within the host (6). MAGs further advance our understanding of taxonomic classification and functional annotation, revealing conserved genes and metabolic pathways across different organisms (7), thus contributing to the expansion of microbial diversity and adaptation (8, 9).

MAGs have been particularly valuable in studying the hosts’ gut microbiota, where many bacteria remain uncultivable due to the difficulty of mimicking intestinal conditions for *in vitro* growth (10). The study of uncultivated microorganisms through MAGs has the potential to uncover novel taxa and contribute to the understanding of microbial roles within host-associated ecosystems (11). Among the biological processes studied in these microorganisms, bacterial sporulation has garnered considerable attention due to its ecological and biotechnological significance (12, 13).

*Firmicutes* (also known as *Bacillota*) is a phylum widely studied in host-microbiota interactions with applications for the immune system and producing beneficial metabolites for the host (11). Within this phylum, *Bacilli* and *Clostridia* classes are known for producing endospores, allowing them to survive extreme environmental circumstances by becoming dormant until conditions become more favorable for growth and development (14, 15). Bacterial spores are metabolically inactive cells contributing to genetic dissemination, adaptation, and promoting bacterial species diversity (16, 17).

Sporulation, typically triggered by nutrient limitation, involves membrane and peptidoglycan remodeling (16). The process begins with the asymmetric division of a vegetative cell into a mother cell and a pre-spore, followed by spore maturation (15, 17–19). Despite the considerable literature on sporulation in cultivated strains, current findings suggest that the ability to form spores may be more widespread within *Firmicutes* than previously believed (20, 21). The adaptability and variability of sporulating bacteria are evident in laboratory cultures, where sporulation loss has been observed in strains of *Bacillus anthracis* and *Bacillus subtilis*, emphasizing their ability to adapt under favorable conditions (22, 23). Despite advances in cultivation techniques (24–26), many intestinal bacteria remain uncultivable under laboratory conditions (27), presenting challenges in characterizing their genetic sporulation repertoire and comprehending their contributions to host health (28, 29).

Detecting sporulation genes in MAGs from host-associated microbiomes enriches our knowledge of bacterial adaptation and their potential implications for health and disease (30, 31). The presence of genetic capacity for sporulation in pathogenic bacteria, often related to diseases such as anthrax, botulism, and infectious diarrhea (32), underscores the importance of investigating the presence of sporulation-associated genes in bacteria residing in hosts and thus contributes to understanding the bacterial persistence and pathogenicity. Conversely, sporulation can also be leveraged for beneficial applications, such as mucosal vaccination and targeted drug-delivery systems in the gastrointestinal tract (15).

The identification of consistent sporulation-related genes in MAGs from multiple hosts contributes not only to developing strategies against sporulation in potentially pathogenic novel species but also to advancing benefit applications by providing new disease prevention strategies and harnessing sporulation mechanisms for innovative biotechnological solutions (33). Here, we investigate the presence of sporulation-associated genes in MAGs obtained from multiple vertebrate hosts, aiming to identify putative novel *Firmicutes* species with potential sporulation capabilities, thus contributing to the ecological significance of sporulation within the *Firmicutes* phylum across different hosts.

## MATERIALS AND METHODS

### Sample collection and bioinformatics analysis

The samples were obtained from the GUARANI One Health Brazilian Group Network and collected between February and April 2020 using swabs in the rectal region of cattle (*n* = 30), swine (*n* = 15), poultry (*n* = 30), and human (*n* = 32, collected from feces) (34). The samples were collected in triplicates from five Brazilian geographic regions: Northern (municipality of Castanhal—Pará/PA state), Southern (municipality of Blumenau—Santa Catarina/SC state), Southeastern (municipality of Bragança Paulista—São Paulo/SP state), Midwestern (municipality of Dourados—Mato Grosso do Sul/MS state), and Northeastern (municipality of Fortaleza—Ceará/CE state). Lemos et al. (34) previously described the steps of DNA extraction, sequencing, and bioinformatics analysis. MAGs were selected based on their completeness and contamination. MAGs of medium quality had completeness ≥50% and contamination <10%, while high-quality MAGs had >90% completeness and <5% contamination (35).

### Selection of genes associated with bacterial sporulation and gene annotation curation

The selection of genes related to bacterial sporulation was based on a recent study that assessed the conservation of these genes in *Firmicutes* (18), a phylum known for its spore-forming ability. In the referenced study, a total of 160 genes associated with ten sporulation processes or regulatory steps, namely Sporulation Onset and Checkpoints (SOAC), Spo0A Regulon, Engulfment, SigF Regulon, SigG Regulon, SigE Regulon, SigK Regulon, Spore Cortex, Spore Coat, and Germination were identified. These genes were selected based on their exclusive or predominant involvement in sporulation with demonstrated roles (18). At the same time, those with known housekeeping functions in vegetative cells were excluded, such as metabolic enzymes and proteins involved in DNA replication, repair, transcription, translation, motility, and secretion, except those involved in cell division, which are also associated with sporulation success (18). Notably, 112/160 genes are universally conserved among *Firmicutes* sporulating members (18). By contrast, 48/160 are mainly conserved within the *Bacilli* class, suggesting a central role in specific characteristics of this taxonomic group (18). The identified genes in our study are listed in Table S1, with the identification of the core components of the bacterial sporulation process. An in-depth analysis of gene annotation data sets was conducted to determine the presence of each gene in MAGs. These data sets included identifiers such as Description (gene-related description), Preferred_name (gene identification), KEGG_ko (KEGG orthology), KEGG_Pathway (KEGG route maps), BRITE (hierarchical classification system in KEGG), and PFAMs (protein family database). Manual curation was performed to standardize the gene annotation information for sporulation-related genes in each MAG from the four vertebrate hosts.

### Identification of taxonomically unassigned MAGs at the family level

The presence of the *spo0A* gene in medium- and high-quality MAGs was used as the first criterion to indicate a microorganism’s potential to initiate sporulation, as this gene has been previously identified as a marker for this process (36, 37). The absence of *spo0A* can prevent bacteria from sporulating (38) and has also been detected in genomes of non-sporulating bacteria (39). Thus, the absence of the *spo0A* gene indicates a non-spore-forming microorganism, while its presence suggests the possibility that the organism sporulates. Initially, the presence of the *spo0A* gene was investigated in each MAG, and those lacking this gene were excluded from subsequent analyses. Since MAGs can be classified to any level of taxonomic information (kingdom, phylum, class, order, family, genus, and species), we selected those without a known taxonomic classification at the family level.

### Presence of sporulation genes in taxonomically unassigned MAGs at the family level

All MAGs were assessed for the presence of each gene associated with sporulation. Following the identification of MAGs containing the *spo0A* gene, the normality of the data corresponding to the frequency of sporulation genes in MAGs from each of the four hosts was verified. For this, the Shapiro-Wilk test (shapiro.test function) (40) was performed using the R programming language (version 4.3.1). Subsequently, to determine whether there was a statistical difference in the number of sporulation genes in all MAGs between hosts, the non-parametric Wilcoxon test (wilcox.test function) (41) was performed with a significance level of 5%, also using the R language. We analyzed the variability profile related to the presence-absence of sporulation-related genes for host and class perspectives using PEMANOVA (adonis2 function) and betadisper function from the vegan package (42), version 2.6.4, by considering 1,000 permutations.

### Reconstruction of phylogenetic trees

We used Graham and collaborators’ phylogenomics workflow to construct phylogenetic trees based on ribosomal proteins (github.com/edgraham/PhylogenomicsWorkflow) (43). While Graham’s original workflow used 16 ribosomal proteins, a new set containing 94 was considered to improve the tree’s accuracy (44, 45). These proteins include universal ribosomal proteins, proteins specific to *Bacteria* or *Archaea*, and proteins specific to taxa, thereby refining the tree’s inference capabilities.

To identify open reading frames (ORFs), we applied the Prodigal v.2.6.3 program (46). Next, a hidden Markov chain algorithm was used to search for ribosomal protein profiles in the genomes of interest. The amino acid sequences were then aligned using MUSCLE v.5.2 (47). Unaligned regions were removed with trimAl v.1.4.22 (48). All ribosomal protein HMM profiles were concatenated for each MAG and the tree inference was performed using FastTree v.2.1.11 (49), applying the Approximately Maximum-Likelihood method and the Le-Gascuel substitution model. We used the iTOL web interface for tree visualization (50), and a bootstrap value of 0.8 was set for the tree analysis (51–53).

The tree reconstruction covered MAGs without taxonomic assignment at the family level (WTAFL) from the GUARANI project, MAGs with taxonomic assignment at the family level containing the *spo0A* gene, and RefSeq/National Center for Biotechnology Information (NCBI) reference genomes with complete assemblies. We used as an outgroup the genomes of the organism *Aquifex aeolicus*. The average nucleotide identity (ANI) between genomes was estimated using the FastANI method, version v.1.33 (54), with a fragment length of 1,020 bp (--fragLen 1020) (55).

Since the taxonomic nomenclature can vary between databases such as Genome Taxonomy Database (GTDB) and NCBI, it was necessary to standardize the nomenclature across databases to obtain reference genomes from NCBI of the same order level from the MAGs. Once the order level was at a higher informative level, the reference genomes were used to complete family-level assignments for the MAGs under study. The conversion was performed directly in the GTDB database by searching for each of the following orders: *Acetivibrionales*, *Bacillales*, *Christensenellales*, *Desulfitobacteriales*, DTU010, *Izemoplasmatales*, *Lachnospirales*, ML615J-28, *Monoglobales*, *Oscillospirales*, RF39, RFN20, *Saccharofermentanales*, *Sporomusales*, TANB77, UBA10575, UBA8346, UBA994, and UMGS1883. These orders were identified as *Eubacteriales*, *Bacillales*, and *Selenomonadales* on the NCBI database. Taxonomic levels for each reference genome were obtained using Unix commands and the Entrez Direct tool to access NCBI databases.

### Functional analysis

Metabolic pathways related to the MAGs were analyzed using the KEGG orthology (KO) available in the EggNOG database, a repository of orthology relationships, functional annotation, and gene evolutionary histories (56). We analyzed the proportion of the genes within each MAG by considering the KEGG level 2 category. Also, for the KEGG level 3 category, we focused on carbohydrate and amino acid metabolism pathways because of their involvement in successful sporulation (57–60), encompassing (i) TCA cycle, (ii) butanoate metabolism, (iii) alanine, aspartate, and glutamate metabolism; (iv) cysteine and methionine metabolism; (v) glycine, serine, and threonine metabolism; (vi) histidine metabolism; (vii) valine, leucine, and isoleucine biosynthesis, (viii) arginine and proline metabolism, and (ix) arginine biosynthesis. To infer the completeness of each of these pathways in the MAGs under study, we used KEGG Orthologies (KOs) of *Bacillus subtilis* (bsu) and *Clostridioides difficile* (cdf) organisms to represent the *Bacilli* and *Clostridia* classes, respectively, because these organisms are models for studying bacterial sporulation (61).

## RESULTS

**Fig 1:**
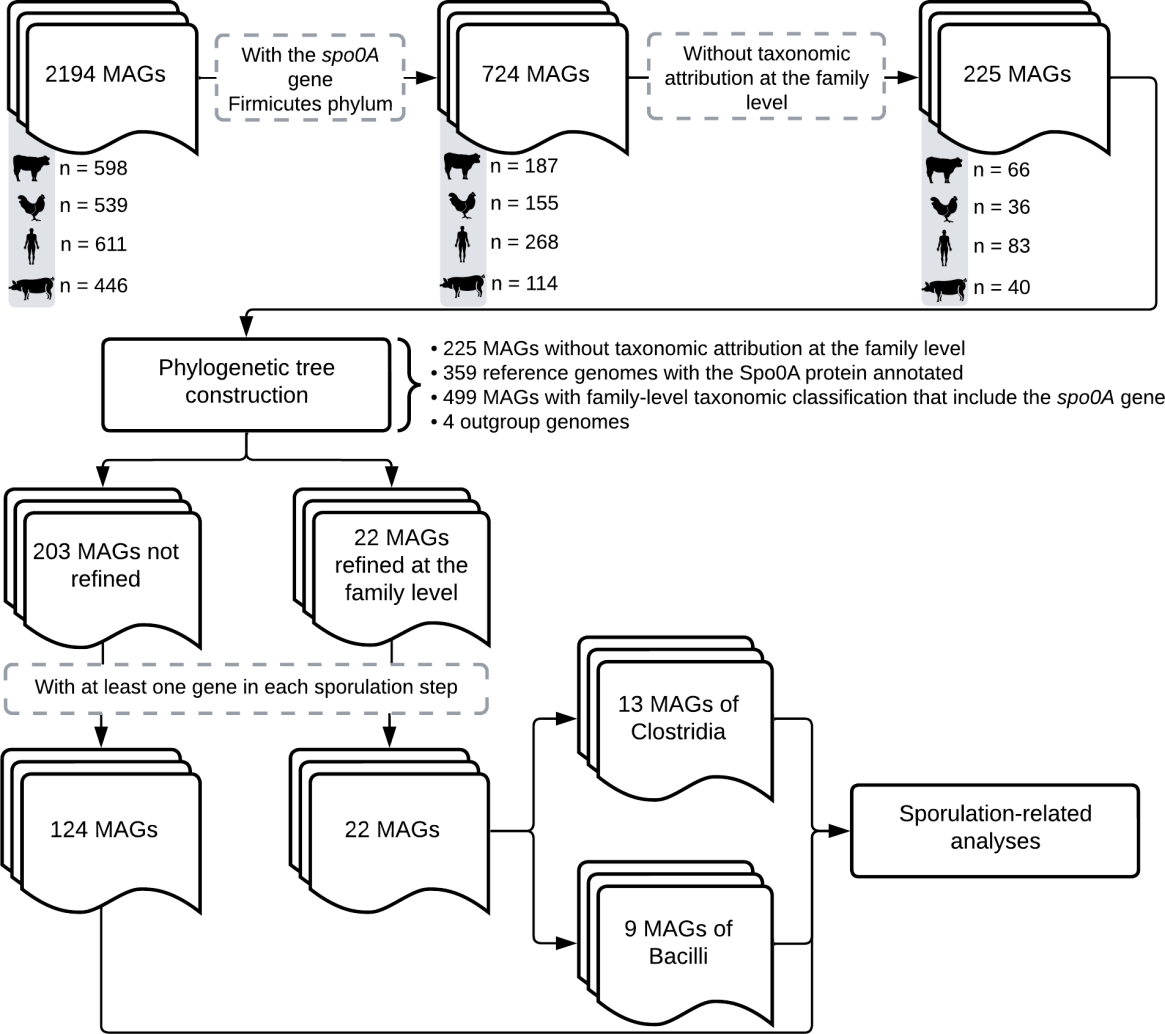
Descriptive summary of the MAGs’ quantity in the analyses realized here.

We summarize the MAG’s quantity throughout the results in Fig. 1. Initially, we examined 2,194 MAGs for the presence of the *spo0A* gene and whether they were part of the *Firmicutes* phylum, resulting in 724 MAGs. Of these, 225 do not have a taxonomic attribution at the family level. After the effort to identify the family using the construction of a phylogenetic tree, we assigned families to 22 MAGs, 13 from the *Clostridia* class, and 9 from *Bacilli*. Although 203 MAGs have not been assigned to a family, we evaluated how many contain at least one gene in each sporulation step, resulting in 124 MAGs. All 22 and 124 MAGs were considered for further sporulation-related analyses.

### MAGs from the phylum *Firmicutes* without taxonomy assignment at the family level are predominantly observed across hosts

**Fig 2:**
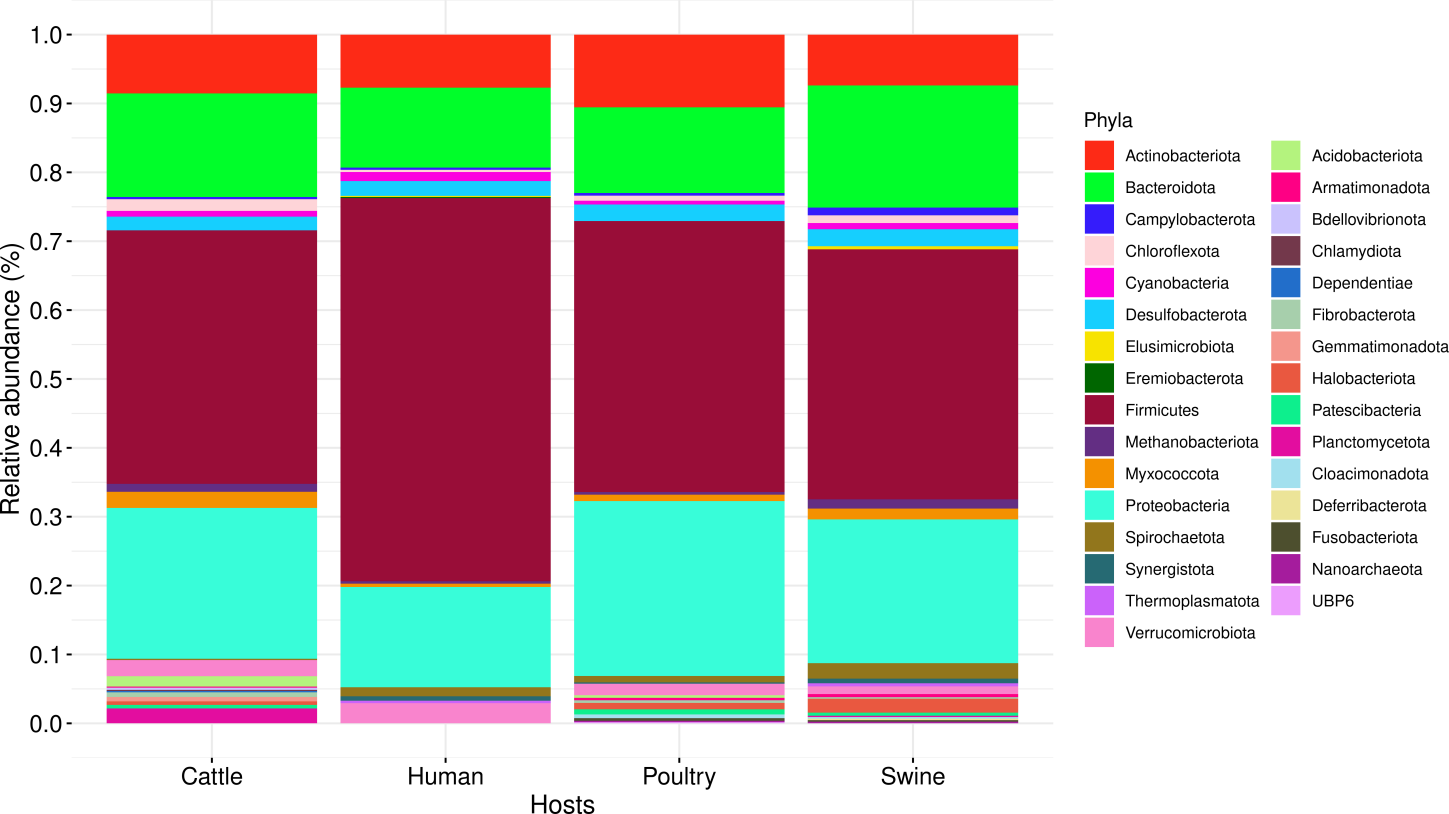
Relative abundance of phyla in MAGs from cattle, humans, poultry, and swine.

Of the total 2,194 MAGs, we identified 611 (27.85%) from humans, 598 (27.25%) from cattle, 539 (24.57%) from poultry, and 446 (20.33%) from swine. In all hosts, MAGs from the phylum *Firmicutes* had the highest relative abundance (~37.48% in cattle, poultry, and swine and 55.65% in humans) (Table S2), followed by *Bacteroidota*, *Proteobacteria*, and *Actinobacteriota* (Fig. 2).

**Fig 3:**
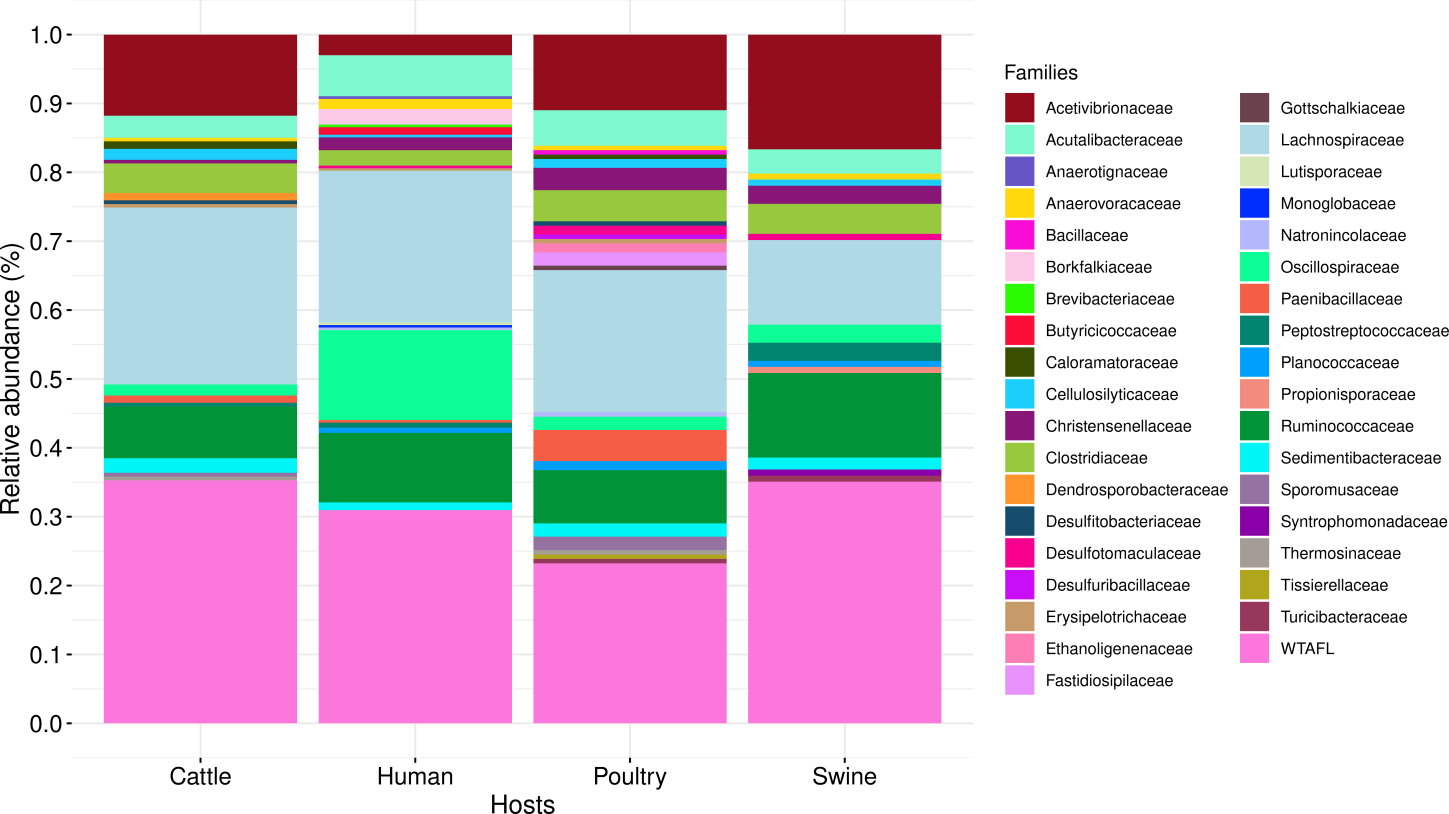
Relative abundance of families associated with MAGs identified in cattle, humans, poultry, and swine. Only MAGs containing the *spo0A* gene were included. WTAFL: without taxonomic assignment at the family level.

In *Firmicutes* phylum, we detected the *spo0A* gene in 724/934 MAGs. Of these, 268 (37.02%) are from humans, 187 (25.83%) from cattle, 155 (21.41%) from poultry, and 114 (15.74%) from swine. Notably, 225/724 MAGs still need to have a well-known family assignment, being identified as MAGs WTAFL, with 83 (36.89%) from humans, 66 (29.33%) from cattle, 40 (17.78%) from swine, and 36 (11.56%) from poultry. These MAGs exhibited the highest relative abundance in all four hosts when compared to those with known families (Fig. 3), with cattle at 35.29%, swine at 35.09%, humans at 30.97%, and poultry at 23.22% (Table S3). The MAGs WTAFL exhibited high completeness and low contamination values (Table S4).

### Taxonomic refinement of 225 MAGs WTAFL

**Fig 4:**
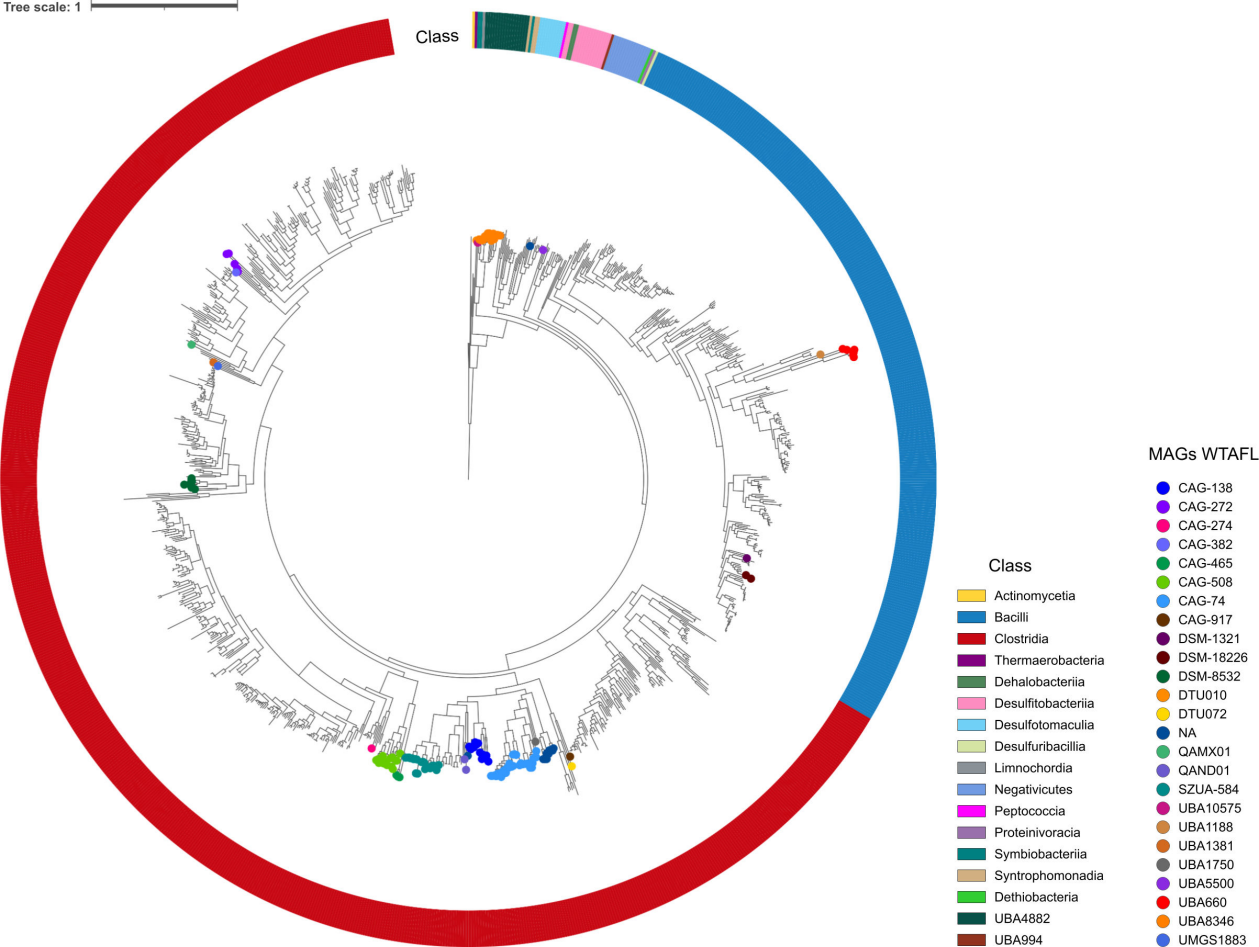
Phylogenetic reconstruction using the Approximately Maximum-Likelihood method and the Le-Gascuel substitution model, based on ribosomal protein sequences, shows the phylogenetic positions of MAGs WTAFL. The 1,087 genomes used encompass reference genomes, MAGs with and WTAFL, and outgroup genomes. MAGs WTAFL, named according to the GTDB database, are indicated on the sheets with colored circles. Information about the classes of these genomes is represented outside the tree.

We constructed a phylogenetic tree to examine the MAGs’ taxonomic position and evolutive relationship within each clade, using the similarity of ribosomal protein HMM profiles (Fig. 4). This analysis included 1,087 genomes. From these, 442 are reference genomes representing three taxonomic orders, namely *Eubacteriales* (15 families and 167 genomes), *Bacillales* (eight families and 269 genomes), and *Selenomonadales* (two families and six genomes) (Fig. S1). Among these genomes, we considered 359 that contained the Spo0A protein, as shown in Fig. S1. Sporulation-related literature for each bacterial family was also considered in this study to examine if MAGs WTAFL are grouping with known sporulating families (Table S5).

Furthermore, 499 genomes with family-level taxonomic classification that include the *spo0A* gene were selected to investigate the evolutionary relationship of MAGs with and WTAFL from the same or different hosts. The tree inference considered all 225 genomes from the MAGs WTAFL category. We refined the taxonomic assignment for 22 MAGs at the family or order level in cases where such classifications were unclear (Fig. 4). Among these 22 MAGs, 13 are classified as *Clostridia* and 9 as *Bacilli*.

**Fig 5:**
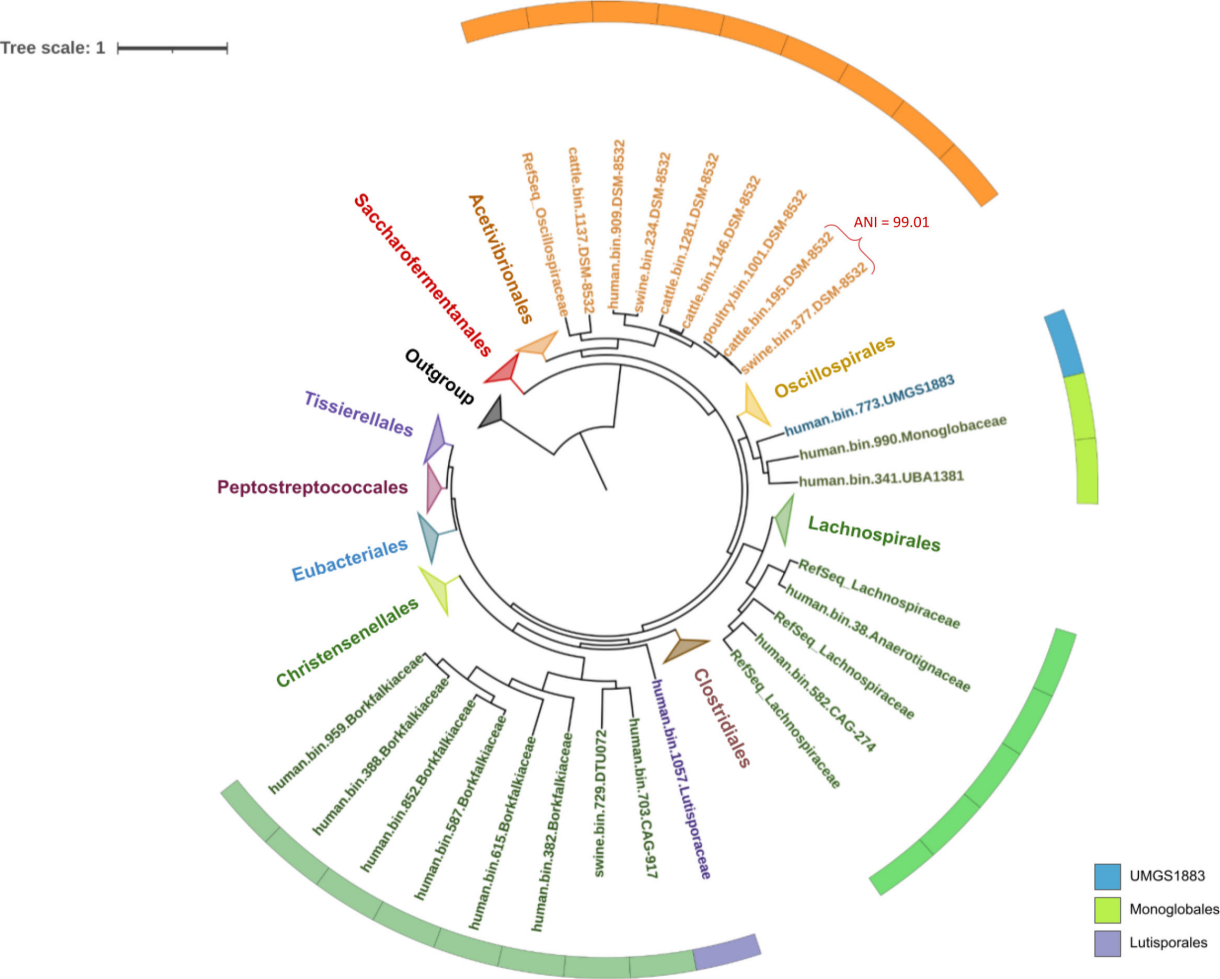
Phylogenetic reconstruction using the Approximately Maximum-Likelihood method and the Le-Gascuel substitution model, based on ribosomal protein sequences of bacterial genomes belonging to the *Clostridia* class. The majority of bacterial orders are identified in the tree’s branches. The remaining ones are in the circle outside the tree.

**Fig 6:**
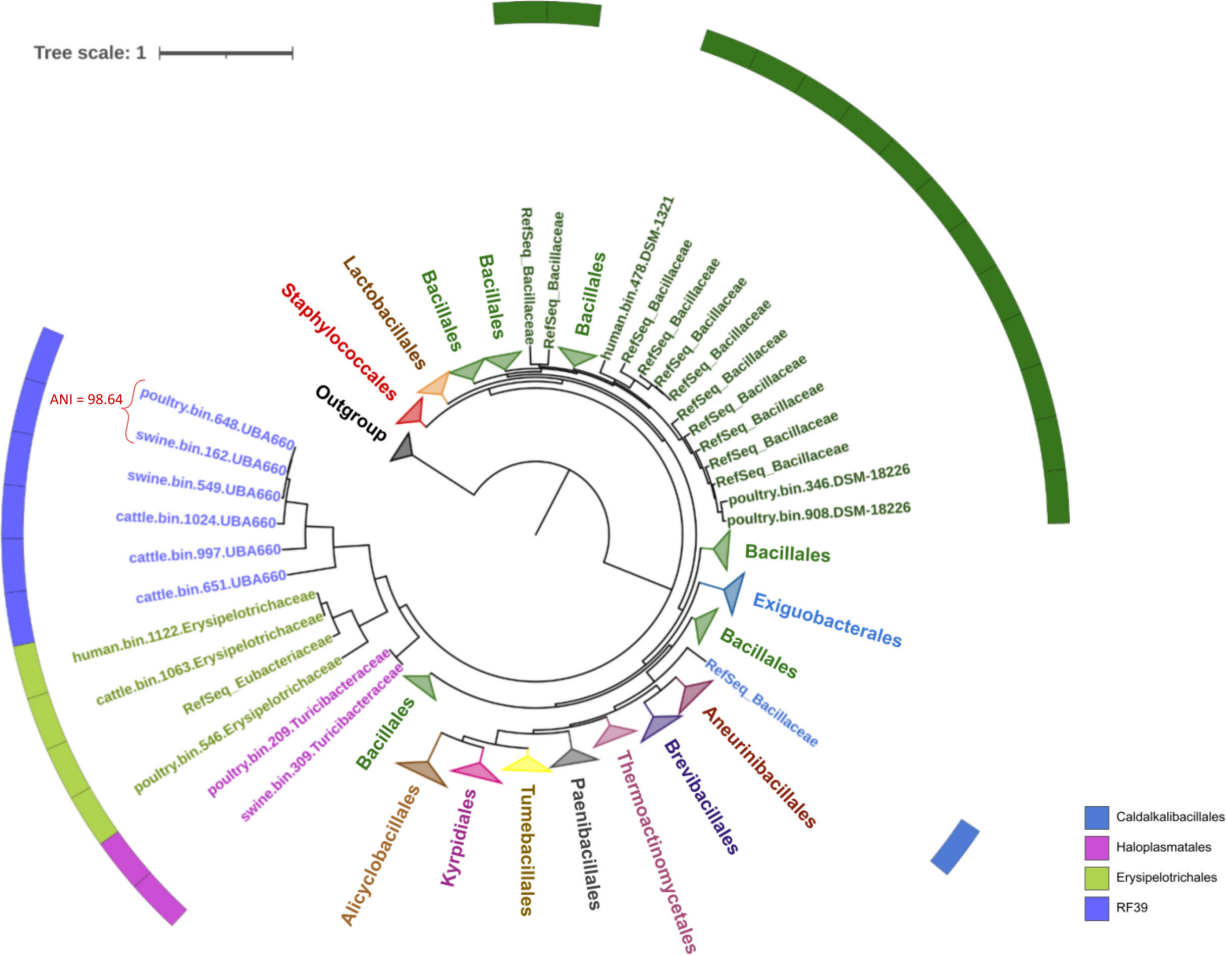
Phylogenetic reconstruction using the Approximately Maximum-Likelihood method and the Le-Gascuel substitution model, based on ribosomal protein sequences of bacterial genomes belonging to the *Bacilli* class. The majority of bacterial orders are identified in the tree’s branches. The remaining ones are in the circle outside the tree.

Based on the tree in Fig. 4, two other phylogenetic trees were constructed for MAGs with assignable taxonomic information at the family or order level: one for the *Clostridia* class (Fig. 5) and another for *Bacilli* (Fig. 6). The first reveals that eight MAGs WTAFL with the suffix -DSM-8532 (cattle.bin.1137, human.bin.909, swine.bin.234, cattle.bin.1281, cattle.bin.1146, poultry.bin.1001, cattle.bin.195, and swine.bin.377) were predominantly positioned in a distant branch descending from the ancestral clade comprised of representatives of the order *Acetivibrionales*. These eight MAGs shared a common ancestor with the *Oscillospiraceae* family (bootstrap = 1), suggesting they could be members of this family (Fig. 5). Notably, two MAGs (cattle.bin.195 and swine.bin.377) exhibited a high ANI value of 99.01, suggesting that both genomes originated from the same species.

Further investigation identified the order-level taxonomy for the MAG human.bin.773.UMGS1883 had a common ancestor with two MAGs in the order *Monoglobales* (human.bin.990.Monoglobaceae and human.bin.341.UBA1381) (bootstrap = 1), supporting the hypothesis that the three MAGs belong to the same taxonomic order (Fig. 5). Interestingly, only one of the two sister MAGs with a known order has a family taxonomic description, identified as *Monoglobaceae* (human.bin.990.Monoglobaceae), which implied in the taxonomic refinement of the second MAG (human.bin.341.UBA1381) to be also a possible member of the *Monoglobaceae* family.

One MAG WTAFL identified as human.bin.582.CAG-274, belonging to the order *Lachnospirales*, is grouped as a sister branch of a reference genome (species *Clostridium colinum*) from the *Lachnospiraceae* family (bootstrap = 1), suggesting probable membership in this family (Fig. 5). In addition, two MAGs WTAFL of the order *Christensenellales* (human.bin.703.CAG-917 and swine.bin.729.DTU072) share a common ancestor with MAGs taxonomic assigned to the *Borkfalkiaceae* family (bootstrap = 1), indicating a close evolutionary relationship between these genomes.

Of the nine MAGs WTAFL belonging to the *Bacilli* class (Fig. 6), three (human.bin.478.DSM-1321, poultry.bin.346.DSM-18226, and poultry.bin.908.DSM-18226) formed a clade with a variety of reference genomes belonging to *Bacillaceae* (bootstrap = 0.99), suggesting that these MAGs could also be members of this family. Moreover, six MAGs with the suffix -UBA660 (poultry.bin.648, swine.bin.162, swine.bin.549, cattle.bin.1024, cattle.bin.997, and cattle.bin.651) were grouped in a clade that shared a common ancestor with the *Erysipelotrichales* clade (bootstrap = 1). However, due to the phylogenetic distance between these MAGs and members of the *Erysipelotrichales*, further in-depth investigation is necessary to understand the conservation of sporulation genes in these genomes. It is important to note that the MAGs poultry.bin.648.UBA660 and swine.bin.162.UBA660 had an ANI value of 98.64, suggesting that both genomes can be from the same species.

### Distribution of sporulating genes in the 225 MAGs across vertebrate hosts

The median quantity of sporulation-associated genes detected in MAGs WTAFL (Table S6) varied significantly in cattle, swine, and poultry compared with those from humans (*P*-value < 0.05) (Fig. S2). Most MAGs WTAFL contains genes associated with each sporulation regulatory step. Notably, the cattle hosts stand out with 51 MAGs, each containing at least one gene related to every one of the ten stages of sporulation considered for this work. Similarly, in the human hosts, there are 42 MAGs with such gene representation; in poultry, 30 MAGs; and in swine, 23 MAGs. Interestingly, 146 MAGs out of the previously mentioned 225 (64.89%) present more genes involved in each regulatory stage of the sporulation process. Of the 146, 22 were refined at the family level, while the remaining 124 could not.

### Analysis of sporulation gene profiles: host and class perspectives in 22 refined family MAGs and 124 non-refined family MAGs

**Fig 7:**
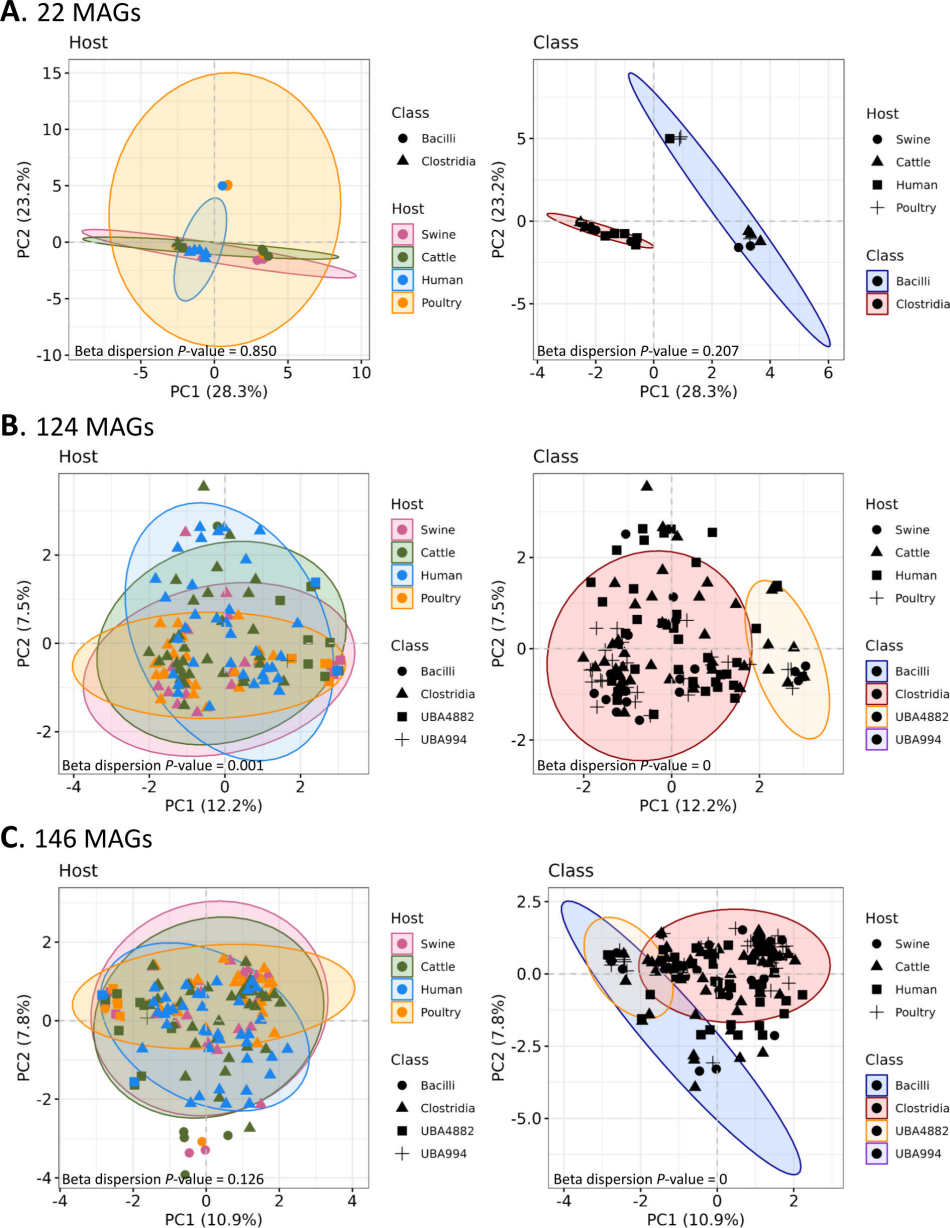
Principal component analysis of the sporulation gene profile across the 22 refined family MAGs (**A**), 124 non-refined family MAGs (**B**), and in these both, totaling 146 MAGs (**C**). Each case represents the influence of host and class as predictor factors for the sporulation profile in MAGs.

We investigated the variability in sporulation gene profiles among hosts and classes using 22 refined family MAGs (classes: *Clostridia* and *Bacilli*) and the remaining 124 MAGs WTAFL group (classes: *Clostridia*, *Bacilli*, UBA4882, and UBA994). For the 22 MAGs (Fig. 7A), the two major components of the PCA analysis explained 51.5% of the variation in sporulation gene presence. Beta dispersion analysis exhibited a *P*-value of 0.850 for hosts and 0.207 for classes, indicating that the gene composition is homogeneous in hosts and also within classes. However, PERMANOVA analysis shows that MAGs belonging to the *Bacilli* class present a different gene sporulation composition than MAGs belonging to *Clostridia* (*P*-value = 0.001). This difference was not observed among hosts (*P*-value = 0.125).

In the group of 124 MAGs without family refinement (Fig. 7B), the two major components of the PCA accounted for 19.65% of the variance in sporulation gene presence. The sporulation gene composition in these MAGs differs within each host (beta dispersion *P*-value = 0.001) and within each class (beta dispersion *P*-value = 0). After combining the data from both sets of MAGs (22 and 124 genomes), the explained variation was 18.74% (Fig. 7C). While genes associated with sporulation were consistent within each host (beta dispersion *P*-value = 0.126), they differed significantly within each class (beta dispersion *P*-value = 0). Thus, PERMANOVA hosts-related analysis indicated that the whole 146 MAGs varied in terms of sporulation genes across hosts. Interestingly, the ellipse of *Bacilli* overlaps with UBA4882, which suggests that the latter has a sporulation composition similar to the *Bacilli*.

### Proportion of sporulation genes among the 22 refined and 124 non-refined family MAGs

**Fig 8:**
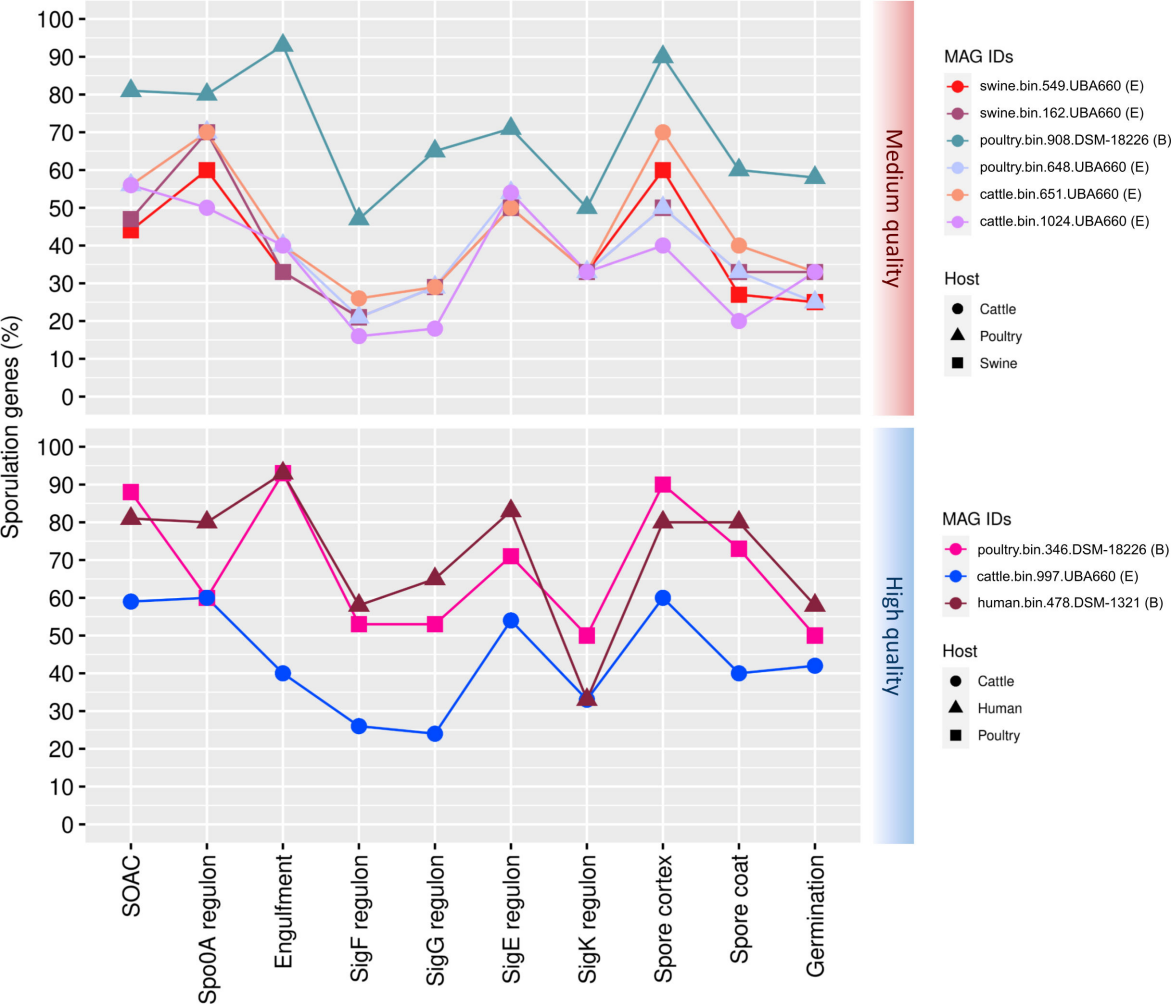
Proportion of genes from each regulatory sporulation stage for nine *Bacilli* MAGs from the 22 refined families. The MAG IDs are identified with family attribution from the phylogenetic tree: (B) for *Bacillaceae* and (E) for *Erysipelotrichaceae*.

After refining the family-level taxonomy of MAGs, we explored the percentage of genes involved in sporulation (Table S7). In the medium-quality group, one MAG from the *Bacilli* class (poultry.bin.908.DSM-18226) presented a higher proportion of sporulation genes than the others (Fig. 8). Two high-quality MAGs (poultry.bin.346.DSM-18226 and human.bin.478.DSM-1321) exhibited the same pattern.

The median of the percentages for each process was computed for each class to which the MAGs are identified. Among the medium-quality MAGs in the *Bacilli* class, Spo0A regulon was predominant, with the presence of 70% of the genes (Table S8). In high-quality *Bacilli* MAGs, the Engulfment step was predominant with 93% of the genes (Fig. 8).

**Fig 9:**
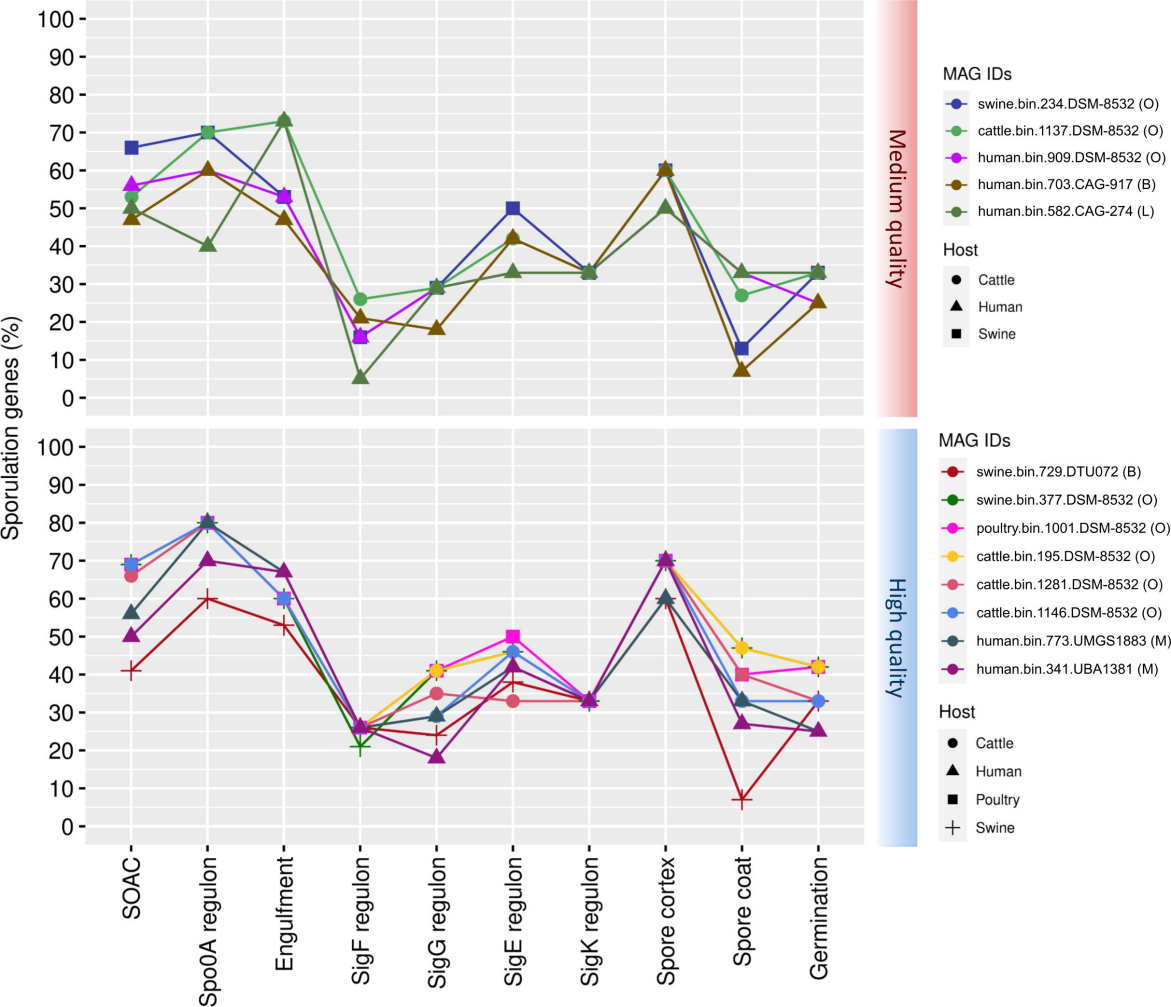
Proportion of genes from each regulatory sporulation stage for 13 *Clostridia* MAGs from the 22 refined families. The MAG IDs are identified with family attribution from the phylogenetic tree: (**O**) for *Oscillospiraceae*, (**B**) for *Borkfalkiaceae*, (**L**) for *Lachnospiraceae*, and (**M**) for *Monoglobaceae*.

For 13 *Clostridia* MAGs, different from the *Bacilli* MAGs, we noticed a similar proportion pattern of the presence of sporulation genes among the medium- and high-quality MAGs (Fig. 9). For medium- and high-quality MAGs, Spo0A regulon was predominant with 60% and 80% of genes, respectively (Table S8).

We expanded the analyses to encompass the 124 MAGs for which the family level was not refined (Table S9). Medium-quality MAGs belonging to the *Clostridia* class had a predominance of the following steps: Spo0A regulon, Engulfment, and Spore Cortex (Table S10), each accounting for 60% of the median gene proportion (Fig. S3). Otherwise, Spo0A regulon was predominant in the high-quality MAGs, accounting for 80% of the median gene proportion. The only high-quality MAG in the *Bacilli* class had the highest gene proportion for the Spo0A regulon, accounting for 50% (Fig. S4). Interestingly, for UBA994 high-quality MAG, the highest gene proportion was at the Engulfment stage, comprising 73% of the genes (Fig. S5). Medium- and high-quality MAGs from the UBA4882 class had the highest median gene proportion at the Engulfment stage (Table S10), representing 67% and 73%, respectively (Fig. S6).

### Consistency of gene presence in *Clostridia* and *Bacilli* family-level refined MAGs

**Fig 10:**
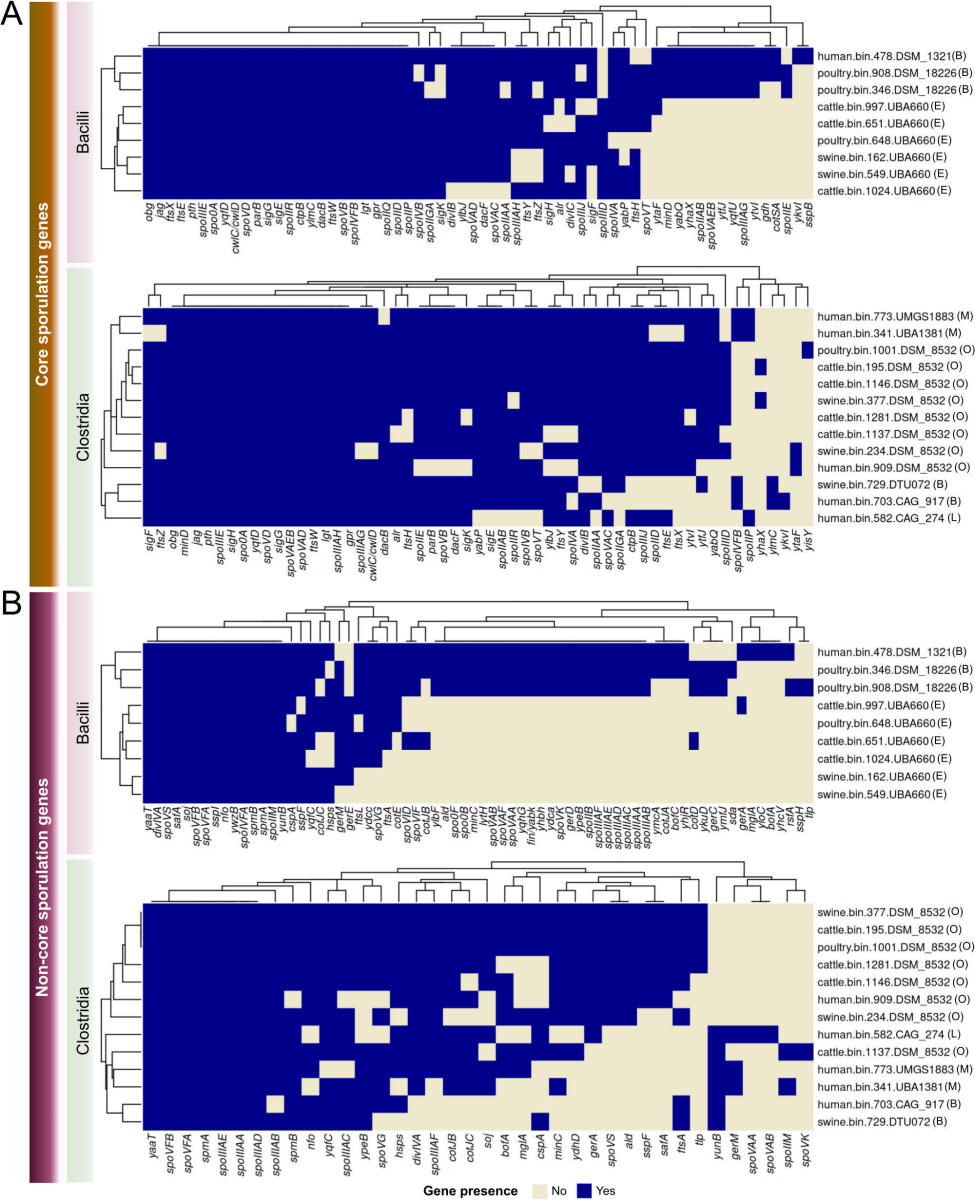
Presence-absence of core (**A**) and non-core (**B**) sporulating genes in the 22 investigated MAGs from *Bacilli* and *Clostridia* classes. The MAG IDs are identified with family attribution from the phylogenetic tree. In *Bacilli*: (**B**) for *Bacillaceae* and (**E**) for *Erysipelotrichaceae*. In *Clostridia*: (**O**) for *Oscillospiraceae*, (**B**) for *Borkfalkiaceae*, (**L**) for *Lachnospiraceae*, and (**M**) for *Monoglobaceae*.

For the 22 MAGs under investigation, we found genes present in those of *Clostridia* and *Bacilli* for each sporulation regulatory step (Fig. 10; Table S11). From those, we identified that 15 genes were present in these two classes, including *jag*, *obg*, *pth*, *spo0A*, *spoIIIE*, and *yaaT* from the SOAC step; *sigG* from the Spo0A regulon; *ftsW* and *spmA* from the SigE regulon; *spoVFA* and *spoVFB* from the SigK regulon; *spoVD* and *yqfD* from spore cortex; and *gpr* and *lgt* from germination. Notably, although the core and non-core sporulation gene quantity did not differ significantly between *Bacilli* and *Clostridia* (Fig. S7), we see a significant difference in non-core sporulating gene quantity among the MAGs of *Clostridia* and UBA4882 (Fig. S8).

In all 146 MAGs, we conducted a correlation analysis between the number of sporulation genes and the genome size (bp). There is a significant positive correlation (r = 0.58, *P*-value = 1.236e-14) representing a tendency for the genome to increase with a greater number of sporulation genes (Fig. S9). Among the 22 MAGs with families identified, the average genome size was 2.327.167 bp (SD ±1.326.052) for the *Bacilli* class. For *Clostridia*, it was 3.378.225 bp (SD ±1.452.479), and for MAGs not taxonomically attributed (NTA) at the family level, it was 3.032.308 bp (SD ±1.290.734).

### Functional analysis in the 22 refined family MAGs and 124 non-refined family MAGs

**Fig 11:**
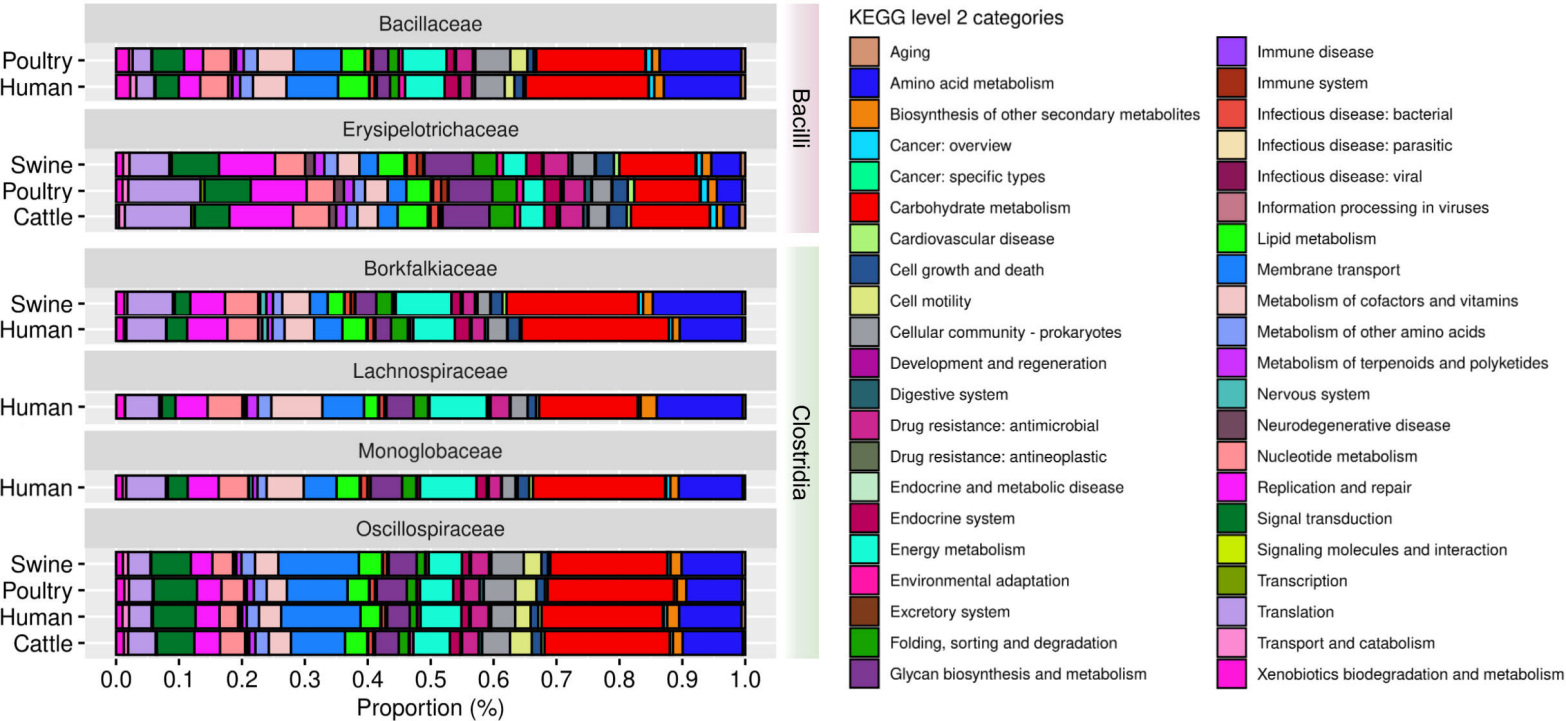
Functional analysis of level 2 KEGG in the 22 MAGs refined taxonomically at the family level.

A comprehensive analysis was conducted on the 22 MAGs refined taxonomically at the family level to investigate the functional characteristics of these microbial genomes. Within the *Bacilli* class (Fig. 11), *Bacillaceae* exhibited carbohydrate metabolism as the predominant category across hosts (Table S12), accounting for an average proportion of 18.42% (SD ±1.50), followed by amino acid metabolism (average proportion equal to 12.61%, SD ±0.52) and membrane transport (average proportion equal to 7.90, SD ±0.42). *Erysipelotrichaceae* showed a carbohydrate metabolism with an average proportion of 11.69% (SD ±1.10), followed by translation (average proportion equal to 9.46%, SD ±2.73) and replication and repair (average proportion equal to 9.29%, SD ±0.69).

In *Clostridia*, carbohydrate metabolism also presented the highest proportions in all families (Fig. 11; Table S12). *Borkfalkiaceae* had an average proportion of this category across the hosts of 22.15% (SD ±1.80), followed by amino acid metabolism (average proportion equal to 12.08%, SD ±3.02) and energy metabolism (average proportion equal to 7.72%, SD ±1.60). The unique MAG of *Lachnospiraceae* accounted for 15.66% of carbohydrate metabolism, 13.64% of amino acid metabolism, and 9.11% of energy metabolism. These three categories were also predominant in the *Monoglobaceae* family, accounting for 21.01%, 10.15%, and 8.96%, respectively. The family *Oscillospiraceae* had the average proportion for carbohydrate metabolism, accounting for 19.45% (SD ±0.68), followed by membrane transport (average proportion equal to 10.92%, SD ±2.12), and amino acid metabolism (average proportion equal to 9.48%, SD ±0.46).

Given the importance of carbohydrate and amino acid metabolism pathways for bacterial germination and outgrowth successes (58), we focused on these categories. Interestingly, they dominated the functional landscape of the 124 MAGs not refined taxonomically (Fig. S10; Table S13). We conducted a detailed investigation into the KEGG level 3 pathways associated with these two categories for the 22 and 124 MAGs.

#### Identification of carbohydrate metabolism pathways in the 22 refined and 124 non-refined family MAGs

**Fig 12:**
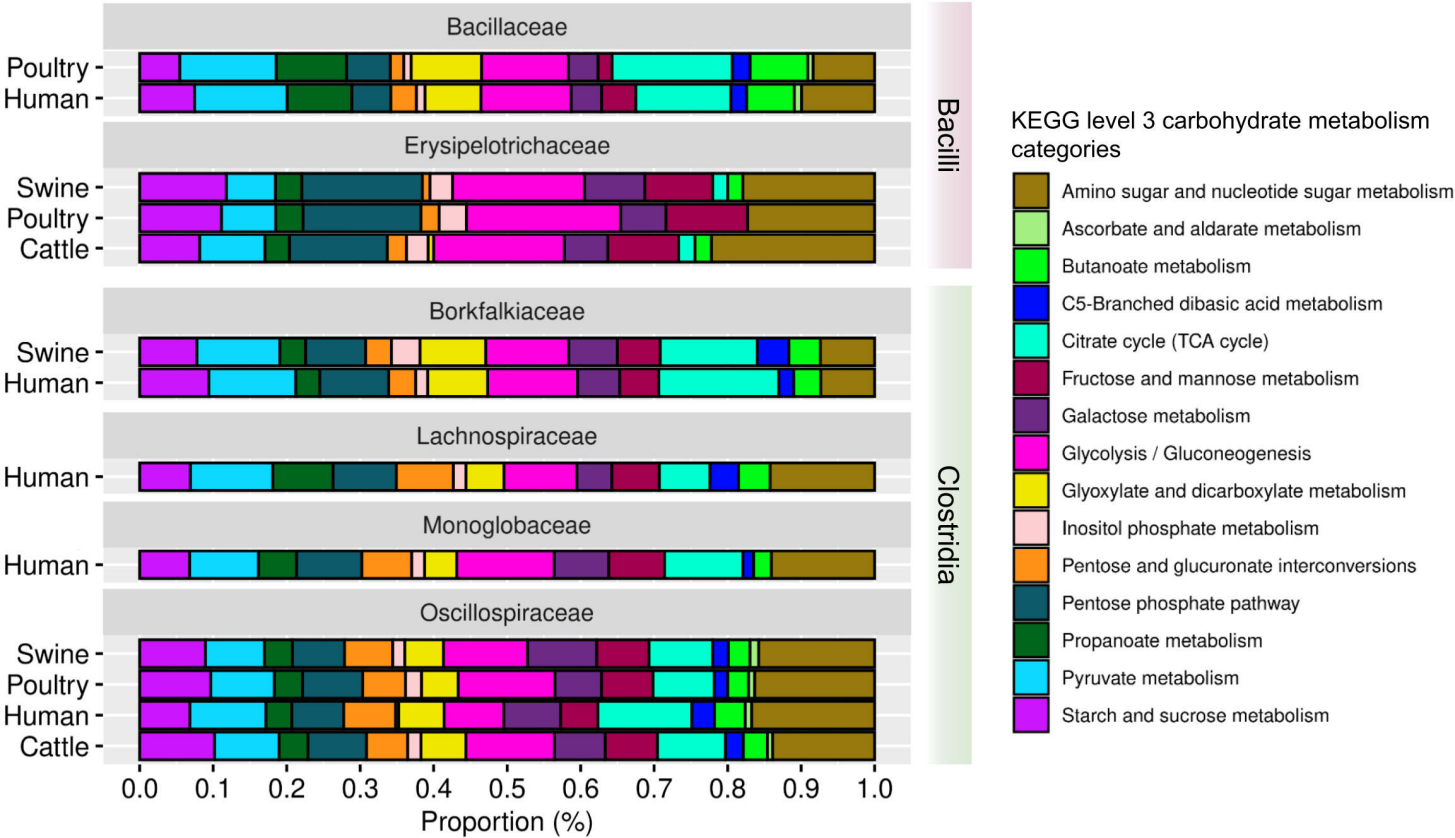
Proportion of carbohydrate metabolism pathways in the 22 MAGs refined taxonomically at the family level.

For the 22 MAGs, we highlight that all of them have genes related to carbohydrate metabolism pathways (Fig. 12; Table S14). The categories (i) amino sugar and nucleotide sugar metabolism and (ii) citrate cycle were in higher proportions among the families. Interestingly, for the 124 MAGs, genes related to glycolysis and gluconeogenesis were present in higher proportions in the *Bacilli*, *Clostridia*, and UBA4882 classes. Otherwise, UBA994 presented a higher proportion in the citrate cycle (Fig. S11; Table S15).

We investigated the presence of bsu and cdf genes related to carbohydrate metabolism in 22 refined and 124 non-refined family MAGs. We focused on the tricarboxylic acid (TCA) cycle and butanoate metabolism for this category. Among the 22 MAGs, *Bacilli* had a higher median gene proportion in the bsu TCA cycle (Fig. S13A). It is worth noting that one high-quality MAG contained 100% of the bsu genes related to the TCA cycle. *Clostridia* MAGs had a higher median gene proportion in the cdf TCA cycle (Fig. S13A). Expanding this analysis to the 124 MAGs, the presence of bsu and cdf genes for carbohydrate metabolism was constant across high- and medium-quality MAGs (Fig. S14A and B).

#### Identification of amino acid metabolism pathways in the 22 refined and 124 non-refined family MAGs

**Fig 13:**
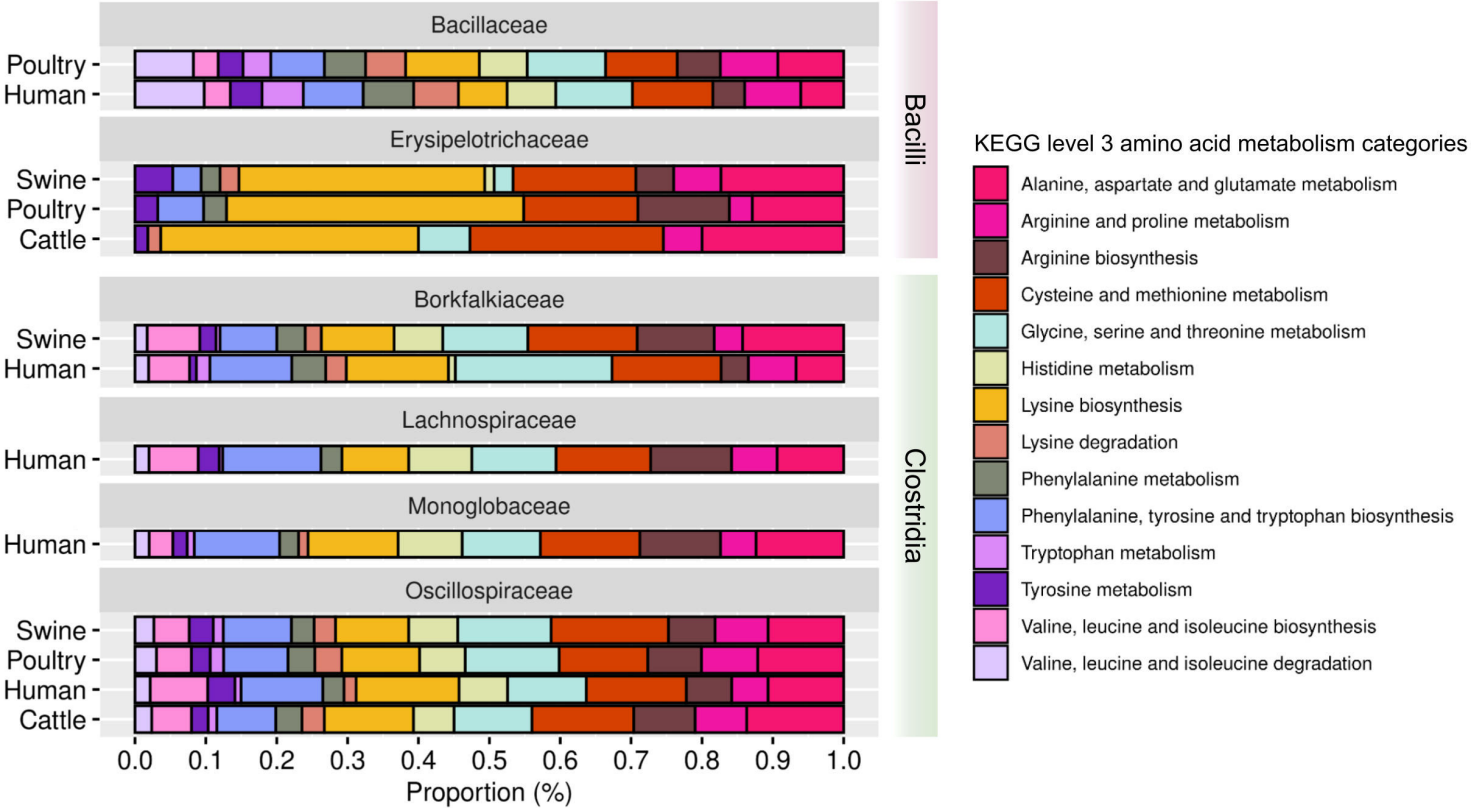
Proportion of amino acid metabolism pathways in the 22 MAGs refined taxonomically at the family level.

In the amino acid metabolism analysis, we highlight the metabolism pathways of glycine, serine, threonine, cysteine, methionine, phenylalanine, tyrosine, tryptophan, and lysine as the higher proportions among the families for the 22 MAGs (Fig. 13; Table S16). For the 124 MAGs, the one from *Bacilli* presented a higher proportion for glycine, serine, and threonine metabolism (Fig. S12; Table S17). MAGs of *Clostridia*, UBA4882, and UBA994 classes presented higher proportions for alanine, aspartate, and glutamate metabolism.

We investigated the presence of bsu and cdf genes related to amino acid metabolism in 22 refined and 124 non-refined family MAGs. We focused on seven amino acid metabolism pathways: (i) alanine, aspartate, and glutamate metabolism; (ii) cysteine and methionine metabolism; (iii) glycine, serine, and threonine metabolism; (iv) histidine metabolism; (v) valine, leucine, and isoleucine biosynthesis, (vi) arginine and proline metabolism, and (vii) arginine biosynthesis.

For the 22 MAGs, *Bacilli* MAGs had a higher median gene proportion in bsu and cdf histidine metabolism (Fig. S13F), bsu and cdf valine, leucine, and isoleucine biosynthesis (Fig. S13G), and bsu and cdf arginine biosynthesis (Fig. S13I). It is worth noting that one high- and one medium-quality MAG presented 100% of bsu genes in histidine metabolism. Two high-quality and one medium-quality MAG presented 100% cdf genes in this pathway. One high-quality MAG presented 100% of bsu genes in bsu valine, leucine, and isoleucine biosynthesis, and also for this pathway, two high- and one medium-quality MAG presented 100% of the cdf genes. One high- and one medium-quality MAG presented 100% of cdf genes in arginine biosynthesis.

Interestingly, *Clostridia* MAGs had a higher median gene proportion for bsu and cdf genes in the following pathways: alanine, aspartate, and glutamate metabolism (Fig. S13C); cysteine and methionine metabolism (Fig. S13D); glycine, serine, and threonine metabolism (Fig. S13E); and for arginine and proline metabolism (Fig. S13H). Remarkably, histidine metabolism cdf genes were 100% present in two high-quality *Clostridia* MAGs (Fig. S13F). Expanding this analysis to the 124 MAGs (Fig. S14C-I), the presence of bsu and cdf genes for amino acid metabolism was similar across high- and medium-quality MAGs.

## DISCUSSION

Here, we investigated the genetic landscape associated with bacterial sporulation in MAGs obtained from vertebrate hosts, including swine, cattle, poultry, and humans. Our exploration focused on MAGs WTAFL, which might represent novel *Firmicutes* species. Through phylogenetic analysis, we successfully identified 22 MAGs belonging to known sporulating families within *Firmicutes*. Furthermore, although 124 MAGs remained unrefined at the family level, we investigated the presence of sporulation-related genes in them, as they could represent genomes from new families within the *Firmicutes* phylum.

The predominance of the *Firmicutes* phylum across the hosts underscores their widespread adaptation. This adaptability is especially relevant given *Firmicutes*’ capacity to produce resistant endospores, facilitating their transmission and impacting host-microbiota interactions (24, 62). Spore-forming bacteria are common in mammalian guts and the sporulation can influence their colonization, persistence, and transmission between hosts (12, 13). Our results align with previous studies that have also identified *Firmicutes* as a dominant phylum in humans (11), poultry (63–66), cattle (67–69), and swine (70–72) suggesting their significant role in host health and ecological balance.

MAGs WTAFL from *Firmicutes* showed consistently higher relative abundance in each host than MAGs with known families. Identifying MAGs WTAFL appears to be common in metagenomic analyses, underscoring the need for further research into their functional analysis and providing information into the roles within specific ecological environments (73–75). Considering that *Firmicutes* members have genes associated with host health homeostasis (11), we proposed identifying which families these MAGs belong to. In addition, we investigated the genetic repertoire associated with bacterial sporulation, as spore-forming bacteria may utilize this mechanism for host organism maintenance.

Our taxonomic refinement efforts assigned MAGs to families within the *Clostridia* and *Bacilli* classes, which are research subjects due to their relevance in human and animal health and their roles in food spoilage (76). The presence of sporulation-related genes in these potentially novel, uncultivable, and spore-forming species emphasizes their significance as targets for future research, including successful isolation and laboratory characterization. Specifically, we identified MAGs within *Clostridia* belonging to *Oscillospiraceae*, *Lachnospiraceae*, *Monoglobaceae*, and *Borkfalkiaceae* families, as well as MAGs in the *Bacillaceae* and *Erysipelotrichaceae* families within *Bacilli*.

The discovery of MAGs within *Oscillospiraceae* suggests the identification of novel sporulating species. As in here, new species have already been found in human feces (77). Moreover, two MAGs with a high ANI value of 99.01, one from swine and the other from cattle indicate the species’ capability to colonize different ecological niches intrinsic to each host. Species recently described in this family can convert glucose into butyric acid, contributing to the food industry (78, 79). Moreover, new species can have implications for developing next-generation probiotics (80). As it is generally accepted that the threshold value for species identification is 95% (81), we suggest that even possibly being new species of *Oscillospiraceae*, the two MAGs could be the same species, such as has been used in literature to describe and propose species belonging to this family, for example (78, 80).

The *Lachnospiraceae* family, known for its spore-forming capabilities within *Firmicutes*, plays a role in the human intestinal microbiota by degrading indigestible macromolecules into short-chain fatty acids (SCFAs) (82, 83), which are involved in anti-inflammatory activities and modulation of the host’s immune system (84). Along with *Oscillospiraceae*, *Lachnospiraceae* have members that produce butyrate, a substance recently proven to enhance *C. difficile* sporulation *in vitro* (57, 85, 86), implying that the identification of novel potential spore-forming species of this family could be the target for further clinical and biotechnological researches. Interestingly, some spore-forming *Lachnospiraceae* members can only be identified using culture-independent methods (87). Thus, the discovered MAG in humans here as part of *Lachnospiraceae* reveals that there still exists an extensive bacterial community to be identified in this well-studied family, and it can contribute to further studies on the functional diversity of the microbiota in species not yet described.

Microorganisms within the *Monoglobaceae* family are known for their ability to degrade pectin, a fiber present in the cell walls of fruits and vegetables (88). The identification and functional study of these microorganisms have contributed to understanding the pectin fermentation in the human colon (88). Thus, identifying new spore-forming bacteria contributes to understanding these organisms’ adaptability within the human intestine and exploring specialized metabolic pathways, offering opportunities to understand their interaction with host health. It is important to note that *Monoglobaceae* has some species described as non-sporulating (89), the same is seen for *Oscillospiraceae* and *Erysipelotrichaceae* (90–94). Although these families have members that undergo sporulation, some of which are very recently described (95–99), the metagenome-based study realized here contributes to future investigations of the evolutionary relationship of the sporulation gene loss in recently described species and undiscovered ones.

The *Borkfalkiaceae* (also known as *Christensenellaceae*) include species with potential immunomodulatory properties beneficial in biotherapies for inflammatory bowel diseases, obesity, and type 2 diabetes (100, 101). Bacteria belonging to *Borkfalkiaceae*, such as *Christensenella minuta* and the recently identified species *Christensenella intestinihominis*, have been described as non-spore forming (100, 102). Therefore, the MAGs under analysis in this study that are grouped with members of *Borkfalkiaceae* may represent novel, potentially sporulating species, contributing to new investigations in studying the sporulation process within this family and its symbiotic interactions with hosts.

Among *Bacilli*, we categorized MAGs as part of the *Bacillaceae* family, known for its focus on sporulating studies. It suggests that still existing novel sporulating species yet to be explored in hosts, which could contribute to further research into their health implications. We also identified MAGs grouping in a clade with members of *Erysipelotrichales*. Still, due to the phylogenetic distance between these MAGs and representatives of *Erysipelotrichales*, further in-depth investigation is necessary to understand the conservation of sporulation genes. It is noteworthy that this order is known to lack genes involved in endospore formation, resulting in a simplified mechanism for engulfment, spore coat, and germination (18, 103), which suggests that it could be a good model to study the mechanisms of sporulation in more detail. Moreover, here we identified two MAGs, one from poultry and another from swine, presenting an ANI value of 98.64, also emphasizing possible sporulating species colonization in different ecological niches.

Interestingly, a recent study by Wu et al. (104) tested different types of commercially available media to successfully isolate strains of *Erysipelotrichaceae* and another study involving uncultivable bacteria of this family identified members with implications for human health (105). To investigate the poultry gut microbiota, for example, strategies have been utilized regarding techniques based on culturing (106, 107). However, these methods can be susceptible to biases and inaccuracies due to the unknown growth requirements of many microorganisms (6). Thus, further metagenomic-based studies could provide a comprehensive functional description in samples from hosts, leading to the successful isolation of possible new species using culture-based approaches.

For sporulation-related analyses, we identified genes present in all 13 MAGs of *Clostridia* and 9 MAGs of *Bacilli* previously identified as important for the sporulation and predominant in all spore formers (18). These genes include *spoIIIAA*, *spoIIIAD*, *spoIIIAE*, *spoIIIAH*, and *spmA* for the *Clostridia* class; *spoIIM*, *spmA*, and *spmB* for the *Bacilli* class. As these genes are related to the engulfment or SigE regulon steps and were identified in MAGs from four different vertebrate hosts, they could be investigated as targets for experimental research about spore control mechanisms in multiple animals.

The quantities of genes associated with sporulation varied significantly among hosts, with cattle, swine, and poultry exhibiting higher median gene quantity than humans. This difference may stem from microorganisms adapting to their hosts by shedding nonessential genes for survival (10, 108–111). The higher sporulation gene quantity in non-human hosts may be related to microorganisms’ adaptation to the human intestine, potentially involving sporulation loss in humans and other host-adaptation strategies such as specialized metabolic profiles and genome reduction (95). Interestingly, once *Firmicutes* spore-forming bacteria can have a minimum genome size of 2.300.000 bp (39), we observed that most of the refined families from *Bacilli* and *Clostridia*, and the remaining MAGs WTAFL, exhibited values above this threshold. A positive correlation between genome size and the number of sporulation genes further supports the sporulation possibility that the MAGs may carry.

While sporulation gene presence is consistent within hosts, significant variations exist between the *Bacilli* and *Clostridia* classes. This disparity has been previously observed, where reference genomes of *Bacilli* and *Clostridia* differed in gene composition for engulfment and spore coat steps (39, 112). This suggests that MAGs exhibiting a sporulation profile similar to either *Clostridia* or *Bacilli* members could follow a similar underlying mechanism of sporulation for both classes. This finding not only reinforces the consistency of the observed difference but also implies a degree of conservation in sporulation steps within the broader *Firmicutes* phylum. Although there is a clear distinction in sporulation profiles when considering the total of MAGs, the classes UBA4882 and *Bacilli* contain MAGs with similar sporulation profiles, suggesting conservation throughout evolution in the UBA4882 unknown class. Further investigation into specific MAGs, such as in the UBA4882 class, could enhance our understanding of their sporulation profiles and classification.

We identified Spo0A regulon, engulfment, and spore cortex as the predominant sporulation steps among the MAGs. Spo0A is a master regulatory protein that controls sporulation initiation in *Bacilli* and *Clostridia* species (113, 114). It binds to specific DNA sequences and regulates the transcription of hundreds of genes involved in sporulation (115). Engulfment is a crucial checkpoint in sporulation that involves cellular rearrangement and is mediated by bacterial proteolytic machinery (116). The formation of the spore cortex during sporulation, constituting the innermost layer of the spore coat, is highlighted for its role in ensuring spore resistance and facilitating germination (15). Consequently, the predominance of these regulatory steps in the investigated MAGs underscores their fundamental importance in shaping the sporulation process within unknown bacterial species, thereby contributing to future investigations aimed at unraveling the microbial life cycles and harnessing their biotechnological potential. Furthermore, when identifying sporulation-associated genes in all 22 MAGs with refined families, the consistent presence of specific genes across all of them suggests their evolutionary significance. It could raise questions about their functional roles in sporulation regulation.

To support the genetic potential of these microorganisms for sporulation, we investigated pathways related to carbohydrate and amino acid metabolism, which provide an energy source for spore formation and are necessary for the success of sporulation (57–60). For bacterial spores to successfully germinate, ATP is stored in the spore progenitor cell (59). Once the spore breaks dormancy and begins the germination phases, it begins the active production of new proteins for the bacteria, requiring stored ATP (117). In addition, carbohydrate and amino acid metabolism pathways have been detected as having differentially expressed proteins during spore outgrowth, implying that they could also be essential for effective germination (58).

Recent studies underscore the energy-intensive nature of sporulation, emphasizing the necessity of a robust energy supply for the proper progression and completion of this process (57–60). By assessing the completeness of these pathways in our 22 refined family-level MAGs and the remaining 124 group, based on KOs from *B. subtilis* and *C. difficile*, we found that the presence of these KOs indicates the potential for these organisms to utilize these pathways during sporulation. This supports that if these organisms undergo sporulation, they could effectively germinate and return to a metabolically active state, as previously discussed.

Our comprehensive analyses of the 22 MAGs with refined family and the remaining 124 MAGs, coupled with the detection of genes related to sporulation and metabolism of carbohydrates and amino acids, contribute to understanding the extent of sporulating species within *Firmicutes* phylum across different host species. Notably, we found genes associated with butanoate metabolism in the investigated families, suggesting potential applications in host health, particularly in butyrate-producing bacteria, as promising probiotics (85). Furthermore, our investigation into the *Erysipelotrichaceae* family revealed a predominance of metabolic genes in poultry feces, as seen previously for this same host (104), reinforcing the importance of understanding the relation of these pathways in spore-forming bacteria from different hosts, its potential therapeutic applications and highlighting the need for novel isolation and culture strategies.

The investigation of the presence of genes associated with bacteria sporulation in this work, together with the *Firmicutes*’ family attribution for MAGs with this taxon uncharacterized, shows that still exists an extensive unexplored community of bacteria that may be participating in some mutualism interaction with hosts. We highlight that, whereas 22 MAGs were refined at the family level, 124 remained without a family when investigated with reference genomes from the same taxonomic order through phylogenetic analysis, raising the possibility that novel families exist within *Firmicutes* that could be investigated. Thus, we propose that future research focusing on the tentative isolation and characterization of novel species across different hosts may uncover new sporulating bacteria within the *Firmicutes* phylum, with implications for their association with host health, industrial applications, and studies on their ability to persist across different hosts environments.

### Conclusions

The identification of possible new species, particularly those capable of sporulation, within *Clostridia* and *Bacilli* families among MAGs WTAFL has important implications for future research, especially on uncultivable organisms and their impact on host health. Here, we identified MAGs as possibly part of the *Borkfalkiaceae*, *Lachnospiraceae*, *Monoglobaceae*, *Oscillospiraceae*, *Bacillaceae*, and *Erysipelotrichaceae* families. *Firmicute*’s ubiquity across hosts demonstrates their adaptation to different ecological niches and their potential role in host-microbiota interactions. Our taxonomic refining efforts have revealed possible new species in the *Clostridia* and *Bacilli* classes, contributing to the unknown microbial diversity. The significant differences in sporulation gene count among hosts and classes can reflect microbial adaptations and evolutionary strategies to different environments. Furthermore, our investigation of functional pathways associated with carbohydrate and amino acid metabolism in MAGs indicates their potential for sporulation and germination, underscoring their importance in microbial life cycles and contributing to potential biotechnology applications. This study emphasizes the necessity of additional research into MAGs from multiple hosts, which could considerably improve our understanding of microbial ecology, eventually leading to advances in human and animal health. While our findings suggest that the *Firmicutes* MAGs from humans, swine, poultry, and cattle hosts have genetic traits associated with sporulation, further experimental testing, including efforts to isolate these species using novel culture media, would be necessary to clarify the sporulation capabilities of potentially novel species. The metagenomic approach used in this work not only enhances our understanding of the spread of sporulation in bacteria from cattle, poultry, swine, and humans, but it can also help in developing novel strategies to control spore-forming pathogens that can be shared by hosts, such as medications that disrupt sporulation or make spores more vulnerable.

## Data Availability

All supplementary tables mentioned in this article are available on the Zenodo repository under the DOI: 10.5281/zenodo.11094059. The data sets are available in the NCBI SRA (www.ncbi.nlm.nih.gov/sra) under BioProject accession number PRJNA684454.

## SUPPLEMENTAL MATERIAL

Figures S1 to S14 spectrum.02113-24-s0001.pdf Supplementary figures depicting analyses involving bacterial sporulation genes and functional analyses.

Supplemental tables spectrum.02113-24-s0002.xlsx Tables S1 to S17.

ASM does not own the copyrights to Supplemental Material that may be linked to, or accessed through, an article. The authors have granted ASM a non-exclusive, world-wide license to publish the Supplemental Material files. Please contact the corresponding author directly for reuse.
