# Supplementary material for: Uncovering new Firmicutes species in vertebrate hosts through metagenome-assembled genomes with potential for sporulation
Source: Microbiol Spectr. 2024 Sep 16;12(11):e02113-24. doi: 10.1128/spectrum.02113-24 (PMC11536998; doi:10.1128/spectrum.02113-24)
Supplement: Figures S1 to S14 — Supplementary figures depicting analyses involving bacterial sporulation genes and functional analyses. [file spectrum.02113-24-s0001.pdf]

Uncovering new *Firmicutes* species in vertebrate hosts through metagenome-assembled genomes with potential for sporulation

Supplementary Figures

Douglas Terra Machado[1], Beatriz do Carmo Dias[1], Rodrigo Cayô[2], Ana Cristina Gales[3], Fabíola Marques de Carvalho[1], Ana Tereza Ribeiro Vasconcelos[1]*

[1] *Laboratório de Bioinformática, Laboratório Nacional de Computação Científica, Avenida Getúlio Vargas 333, Quitandinha Petrópolis, Rio de Janeiro, 25651-075, Brazil*

[2] *Laboratory of Environmental Antimicrobial Resistance (LEARN), Departamento de Ciências Biológicas (DCB), Instituto de Ciências Ambientais, Químicas e Farmacêuticas (ICAQF), Universidade Federal de São Paulo (UNIFESP), Unidade José Alencar - Rua São Nicolau, 210 (Sala 04 - 1º andar), Centro, Zip Code: 09913-030, Diadema - SP, Brazil.*

[3] *Laboratório ALERTA, Division of Infectious Diseases, Escola Paulista de Medicina (EPM), Universidade Federal de São Paulo (UNIFESP), Rua Pedro de Toledo (6º andar fundos), 781, Vila Clementino, Zip Code: 04039-032, São Paulo - SP, Brazil.*

* Corresponding author: Ana Tereza Ribeiro Vasconcelos
Email: atrv@lncc.br

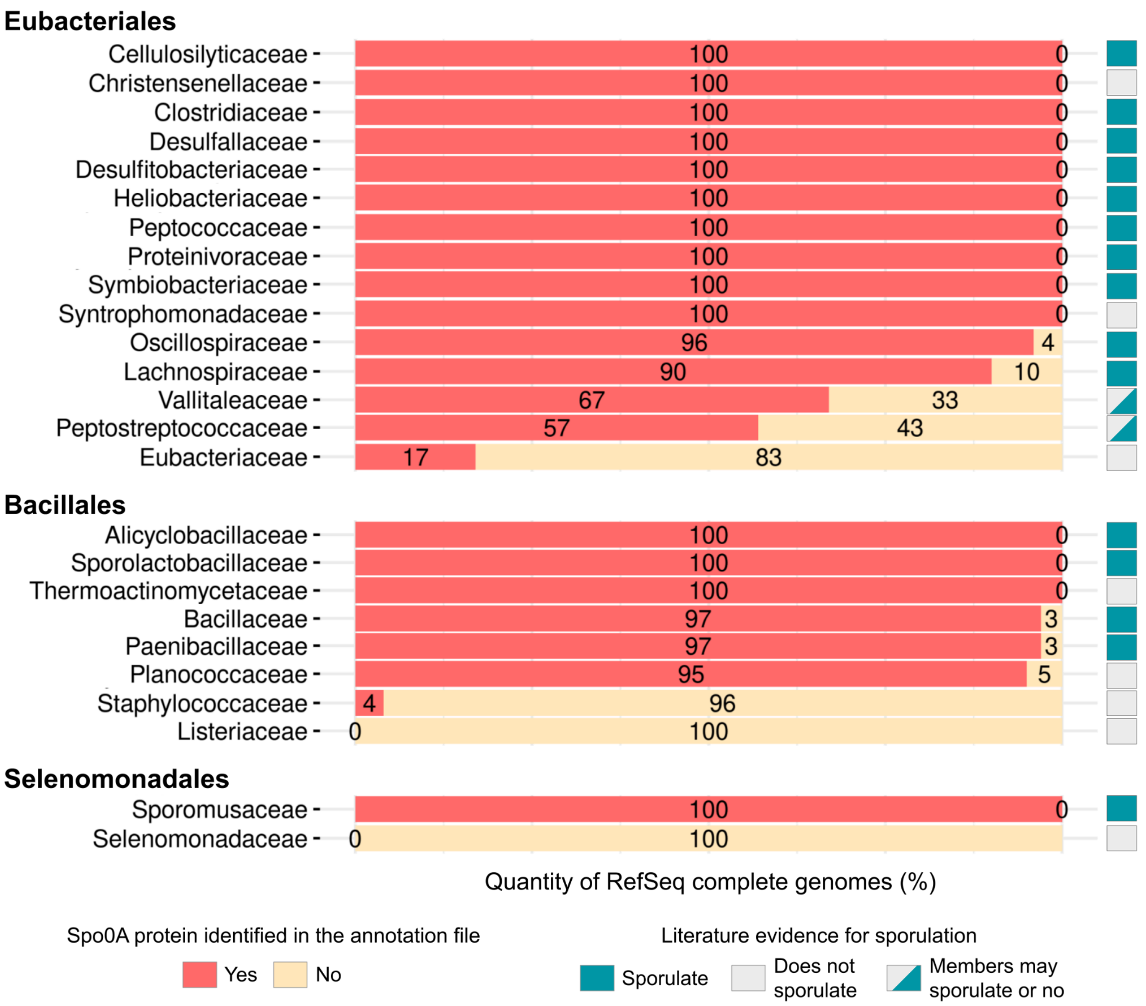

Supplementary Figure 1. Proportion of reference genomes from the orders *Eubacteriales*, *Bacillales*, and *Selenomonadales* used to construct the phylogenetic tree. The presence of the Spo0A protein is identified in the annotation file of each genome. Additionally, literary evidence is represented about whether or not the listed families have individuals known to undergo sporulation.

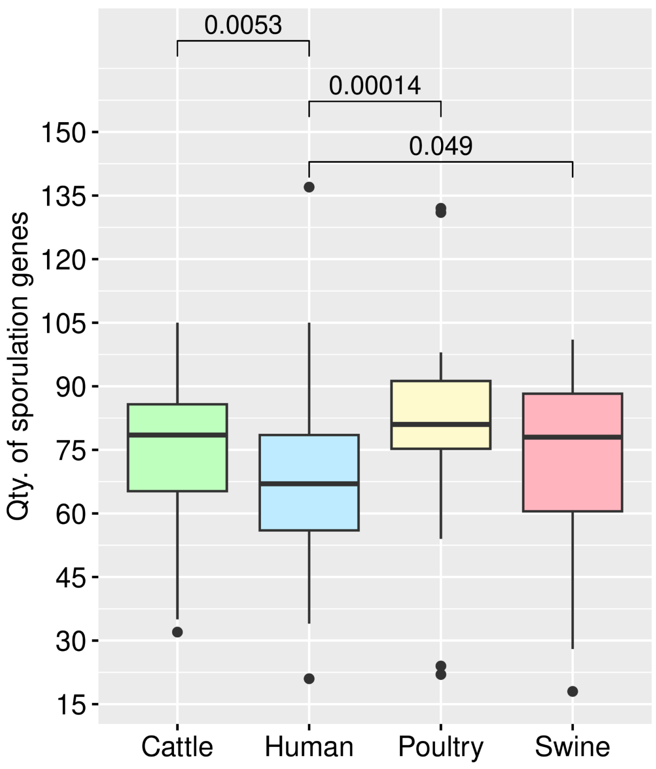

Supplementary Figure 2. Comparative analysis of gene sporulation quantities in 225 MAGs WTAFL from cattle, poultry, humans, and swine. Statistical significance is shown in the difference between cattle and human, human and poultry, and human and swine.

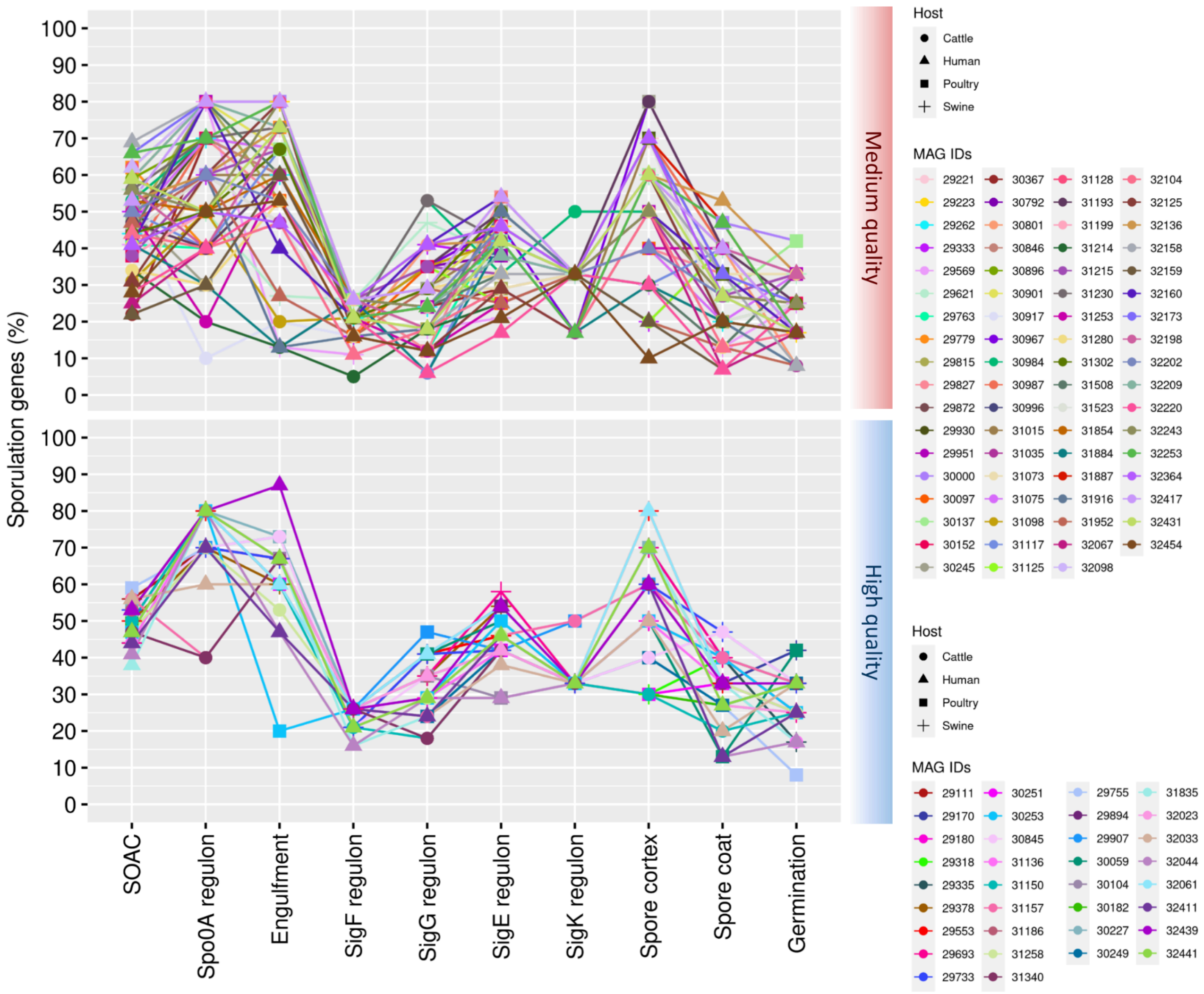

Supplementary Figure 3. Proportion of genes from each regulatory sporulation stage for 105 *Clostridia* MAGs from the 124 unrefined families.

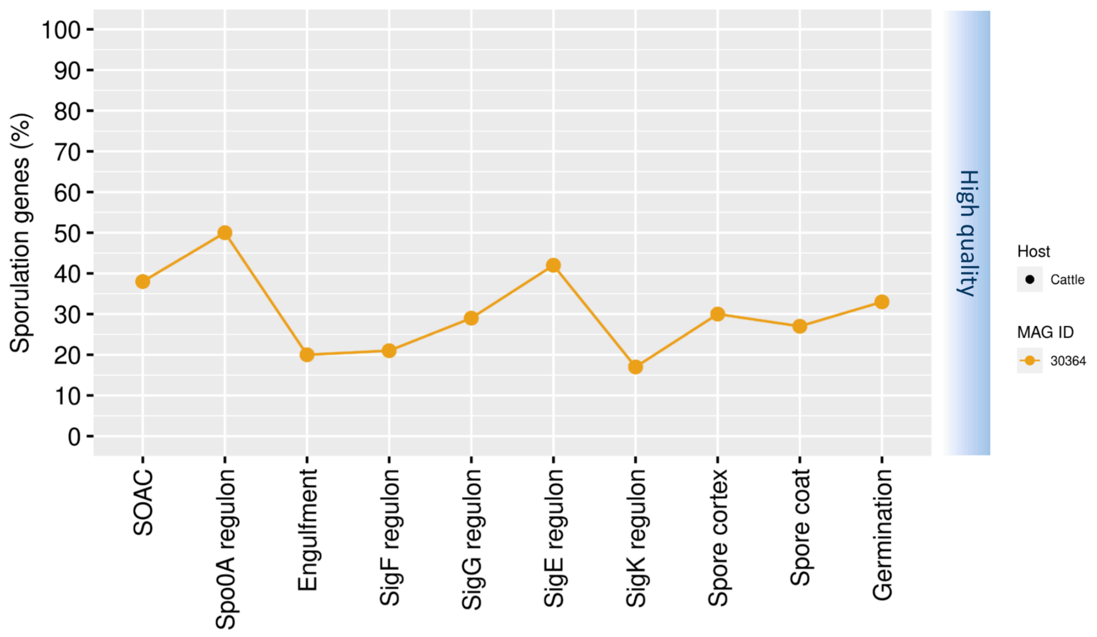

Supplementary Figure 4. Proportion of genes from each regulatory sporulation stage for one *Bacilli* MAG from the 124 unrefined families.

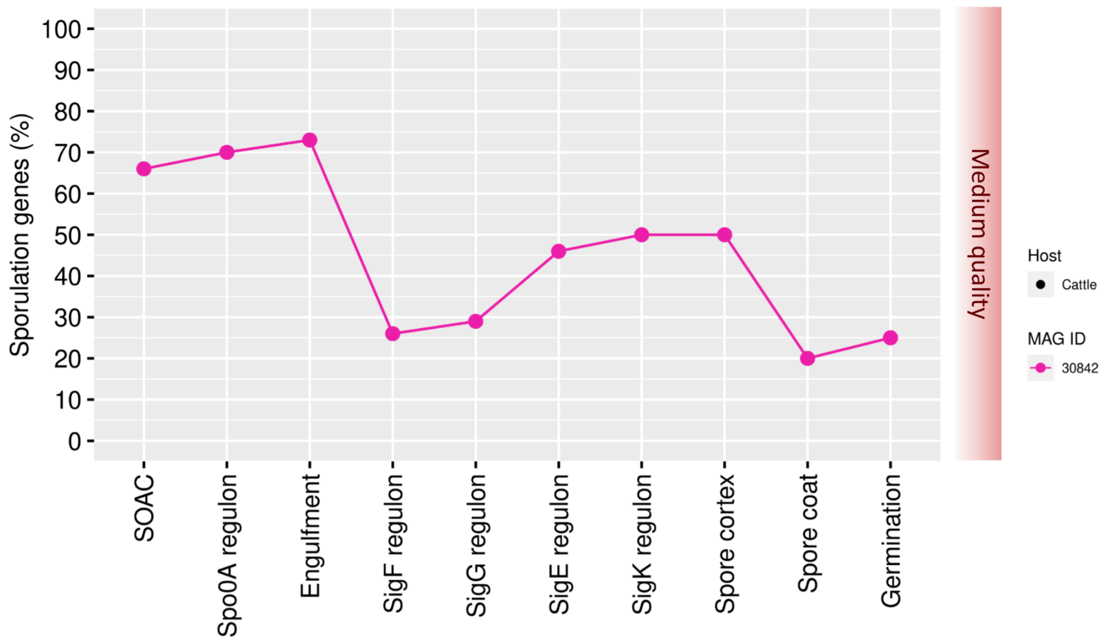

Supplementary Figure 5. Proportion of genes from each regulatory sporulation stage for one UBA994 MAG from the 124 unrefined families.

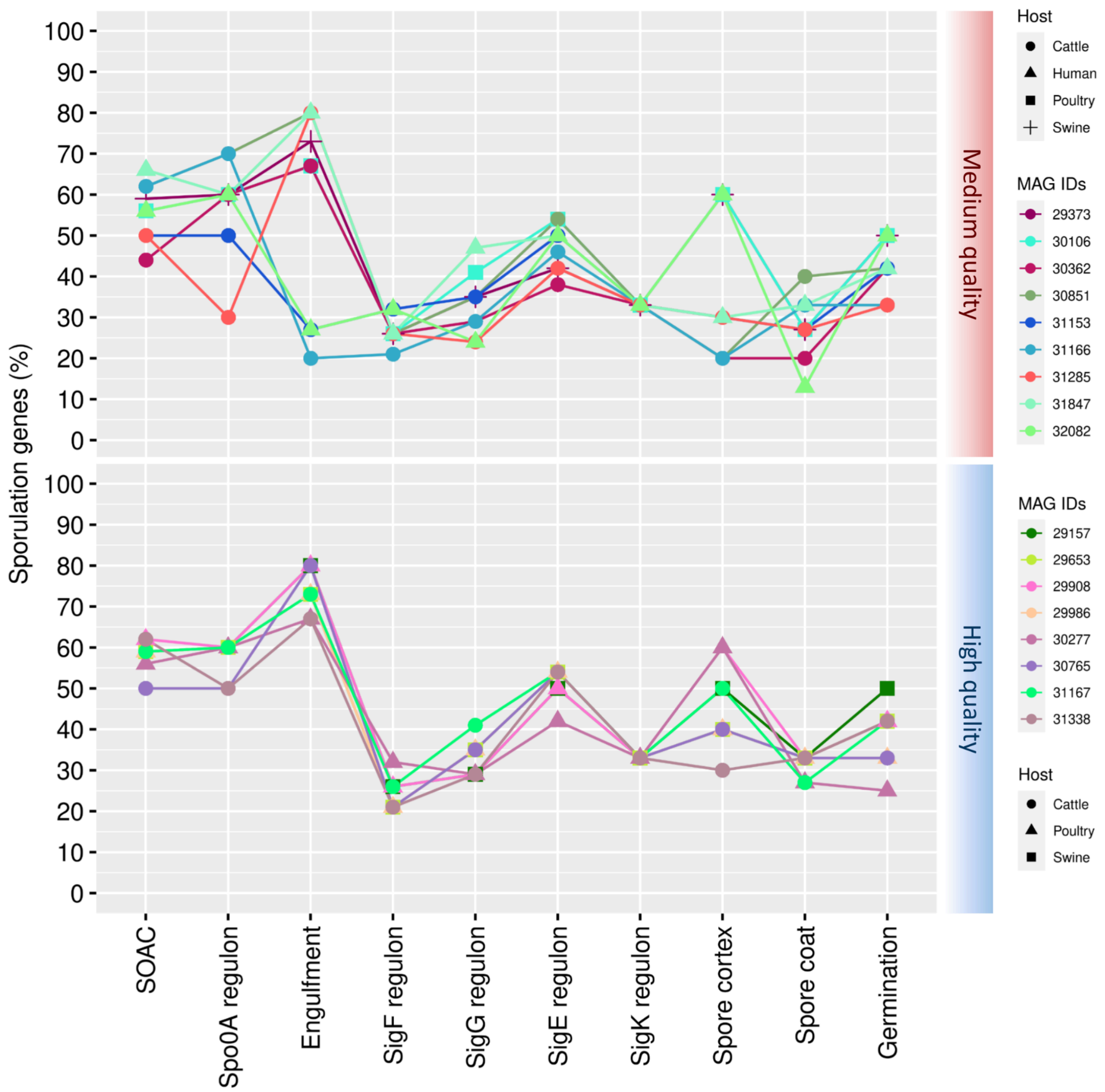

Supplementary Figure 6. Proportion of genes from each regulatory sporulation stage for 17 UBA4882 MAGs from the 124 unrefined families.

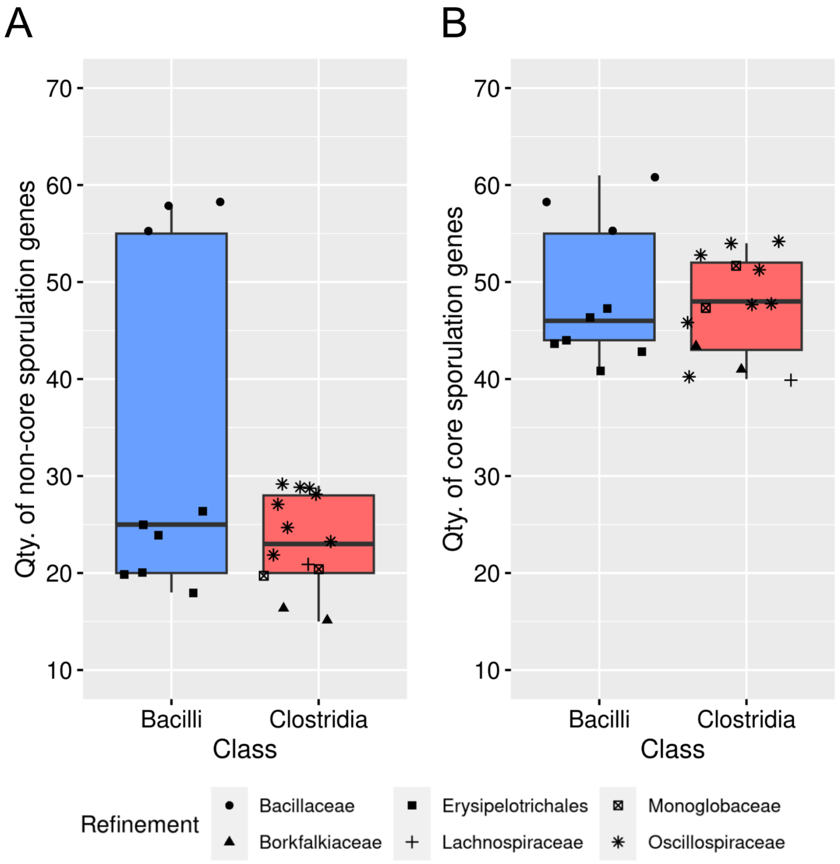

Supplementary Figure 7. Quantity of sporulation genes in the 22 MAGs taxonomically refined at the family level.

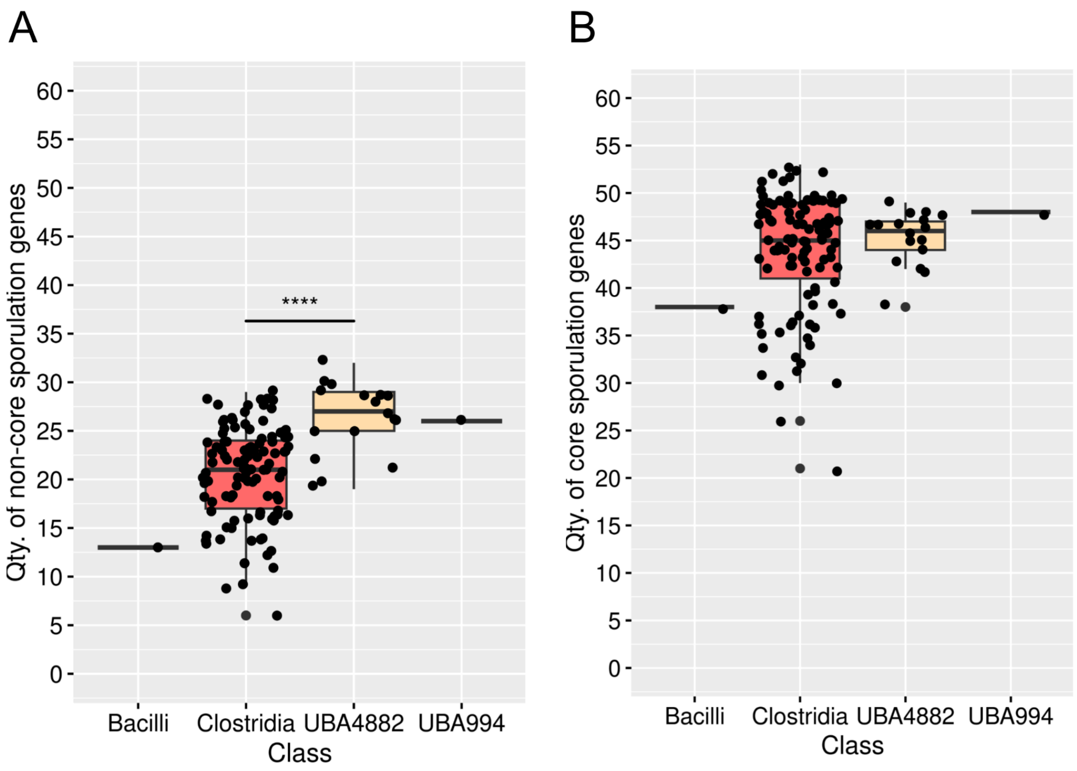

Supplementary Figure 8. Quantity of sporulation genes in the 124 MAGs non-taxonomically refined at the family level.

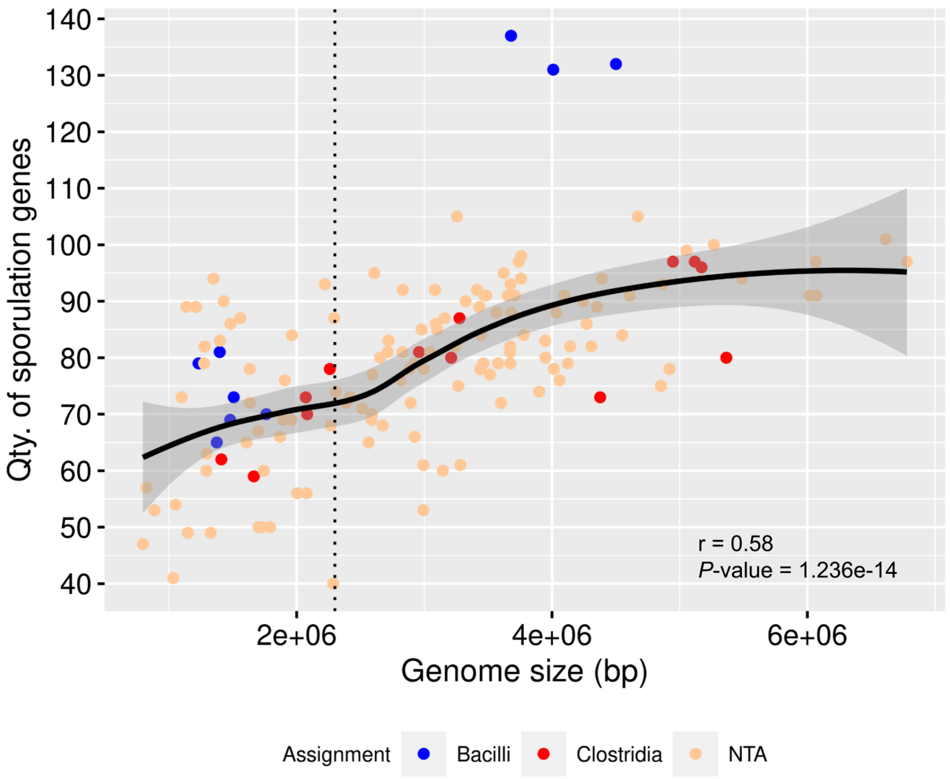

Supplementary Figure 9. Correlation between the genome length (bp) and the quantity of sporulation genes in the 146 MAGs. Blue dots are *Bacilli* MAGs, red dots are *Clostridia* MAGs, both corresponding to the 22 MAGs with families identified, and orange dots are the not taxonomically attributed (NTA) MAGs at the family level.

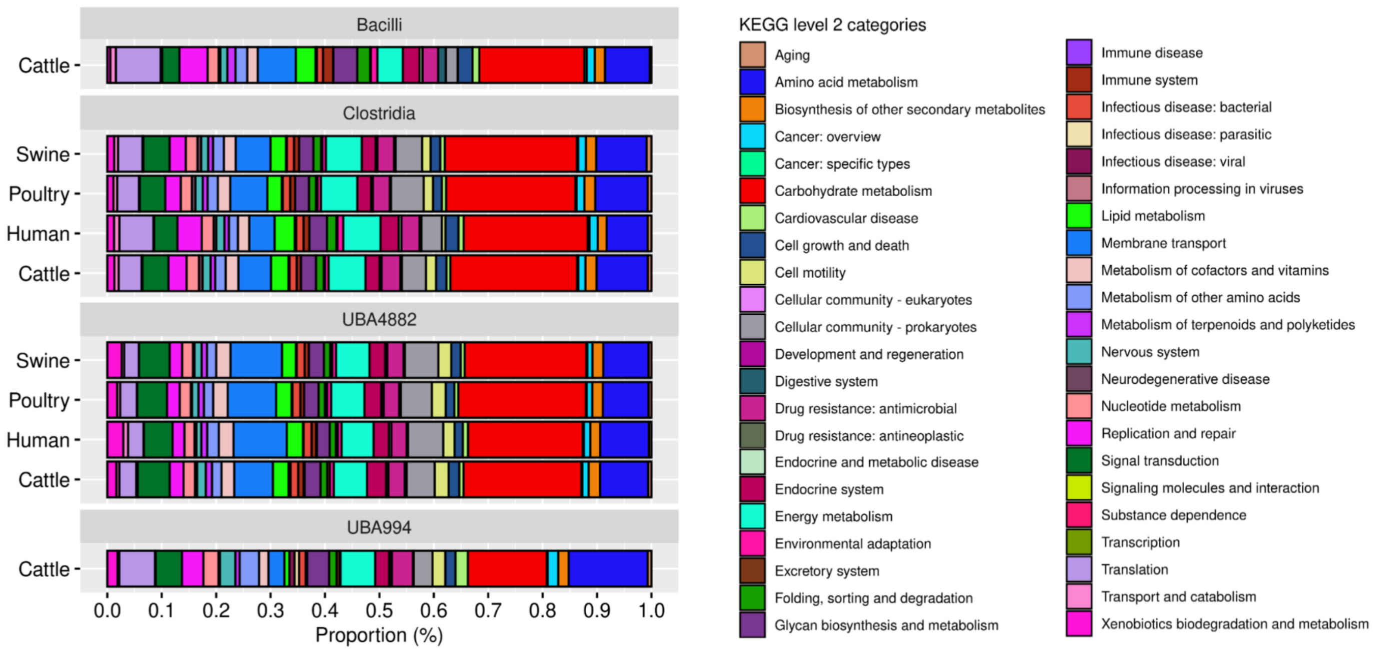

Supplementary Figure 10. Functional analysis of level 2 KEGG in the 124 MAGs not refined taxonomically at the family level.

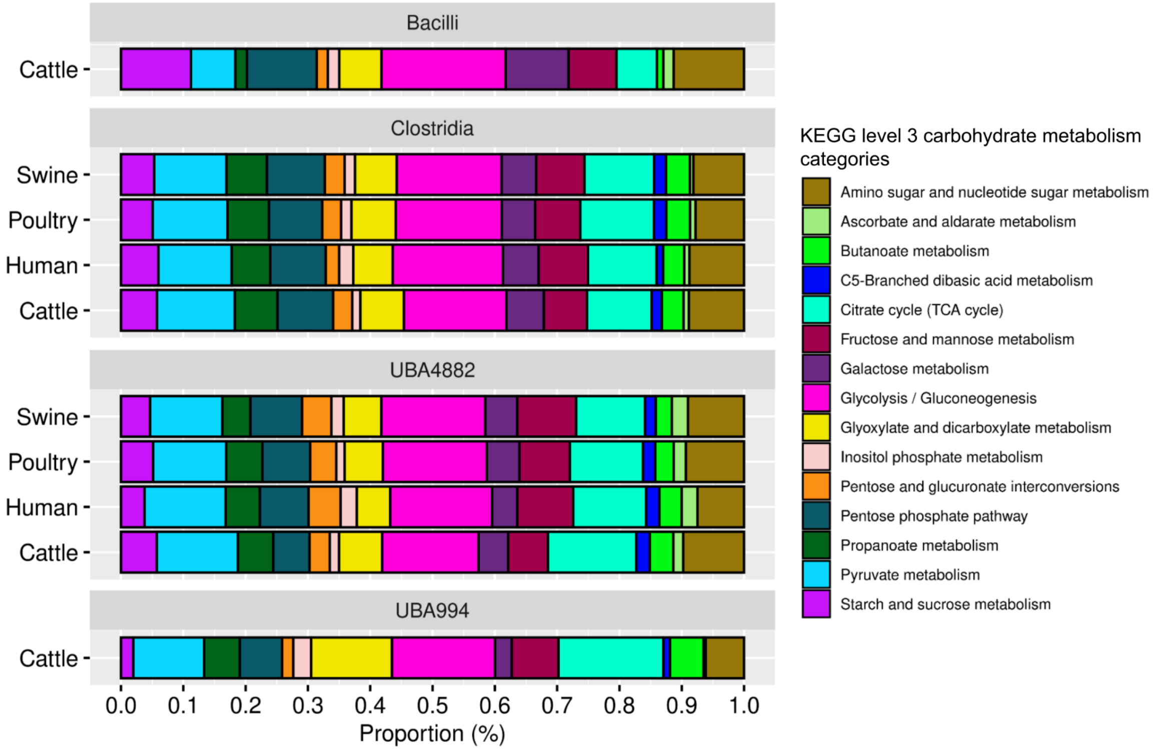

Supplementary Figure 11. Proportion of carbohydrate metabolism pathways in the 124 MAGs not refined taxonomically at the family level.

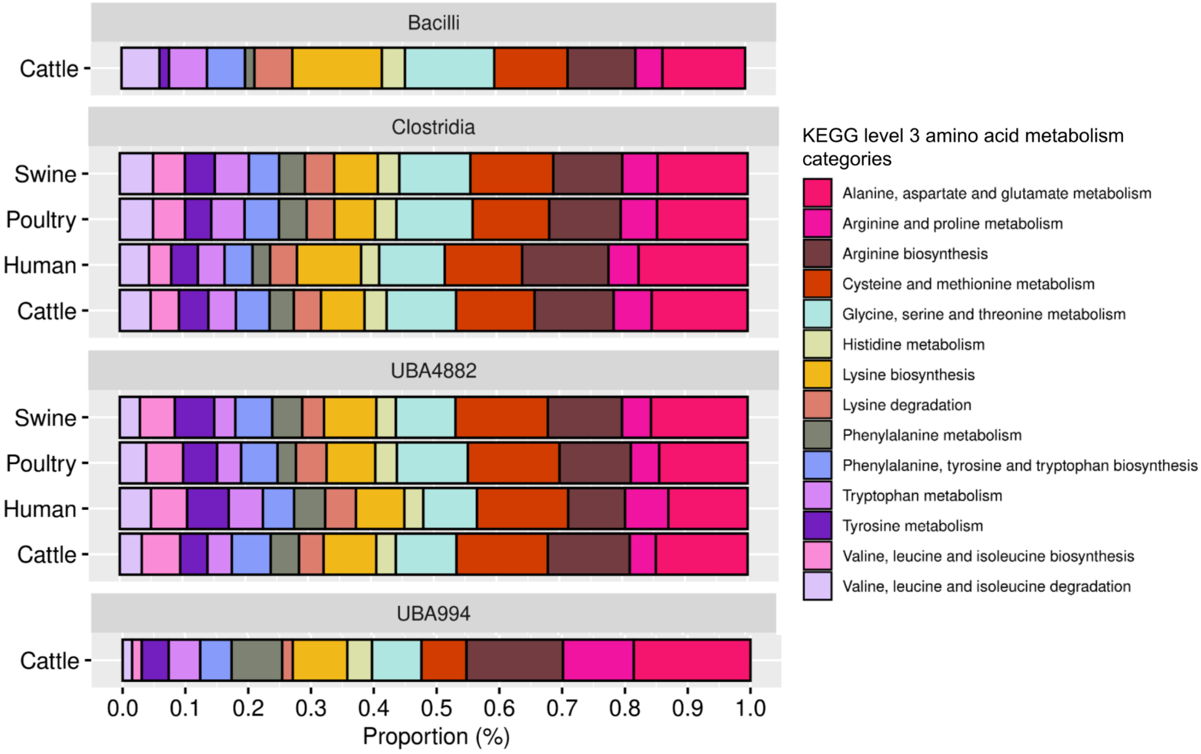

Supplementary Figure 12. Proportion of amino acid metabolism pathways in the 124 MAGs not refined taxonomically at the family level.

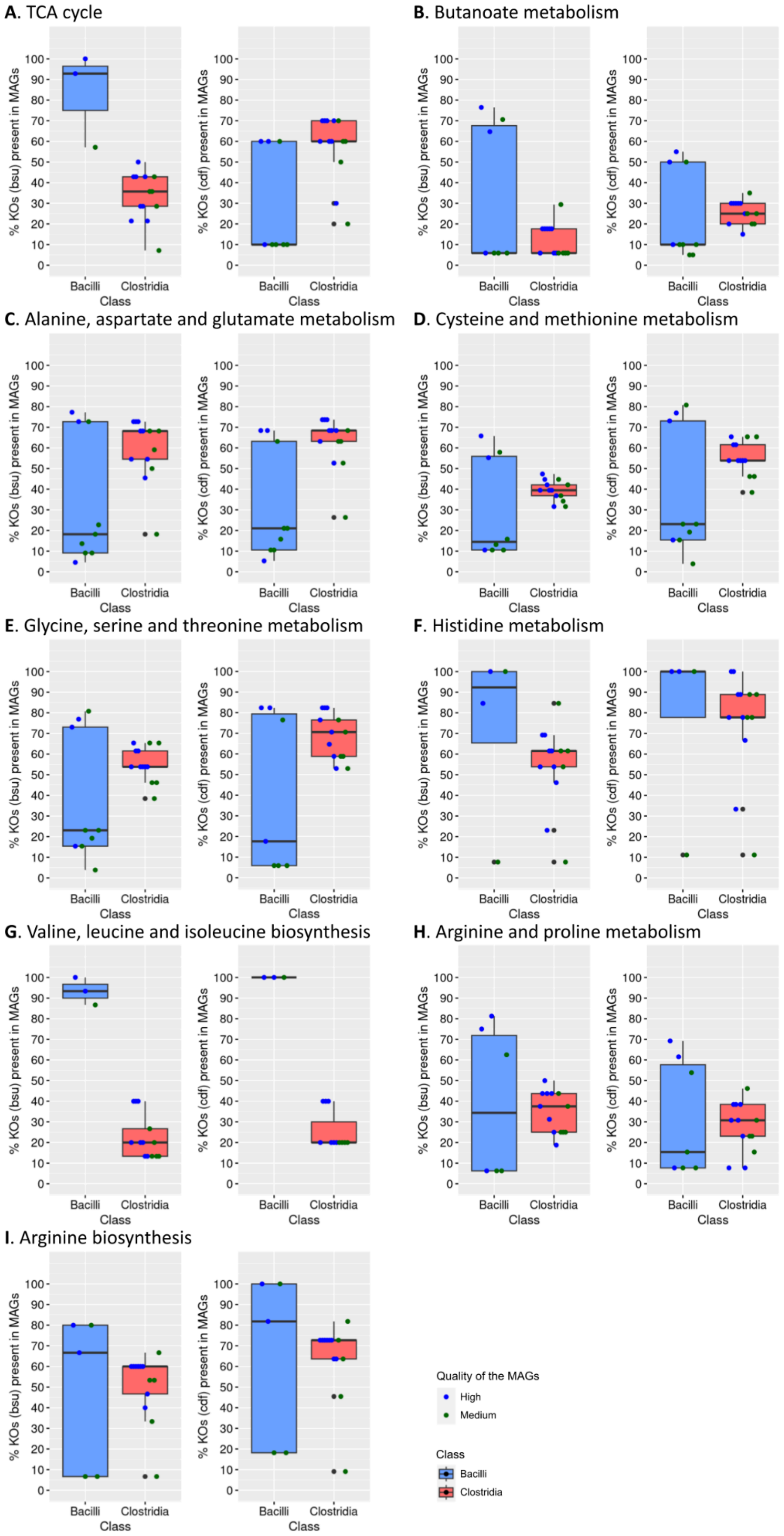

Supplementary Figure 13. Proportion of KOs related to carbohydrate and amino acid pathways in the 22 MAGs refined taxonomically at the family level. A and B are carbohydrate metabolism pathways, and C-I are amino acid metabolism pathways.

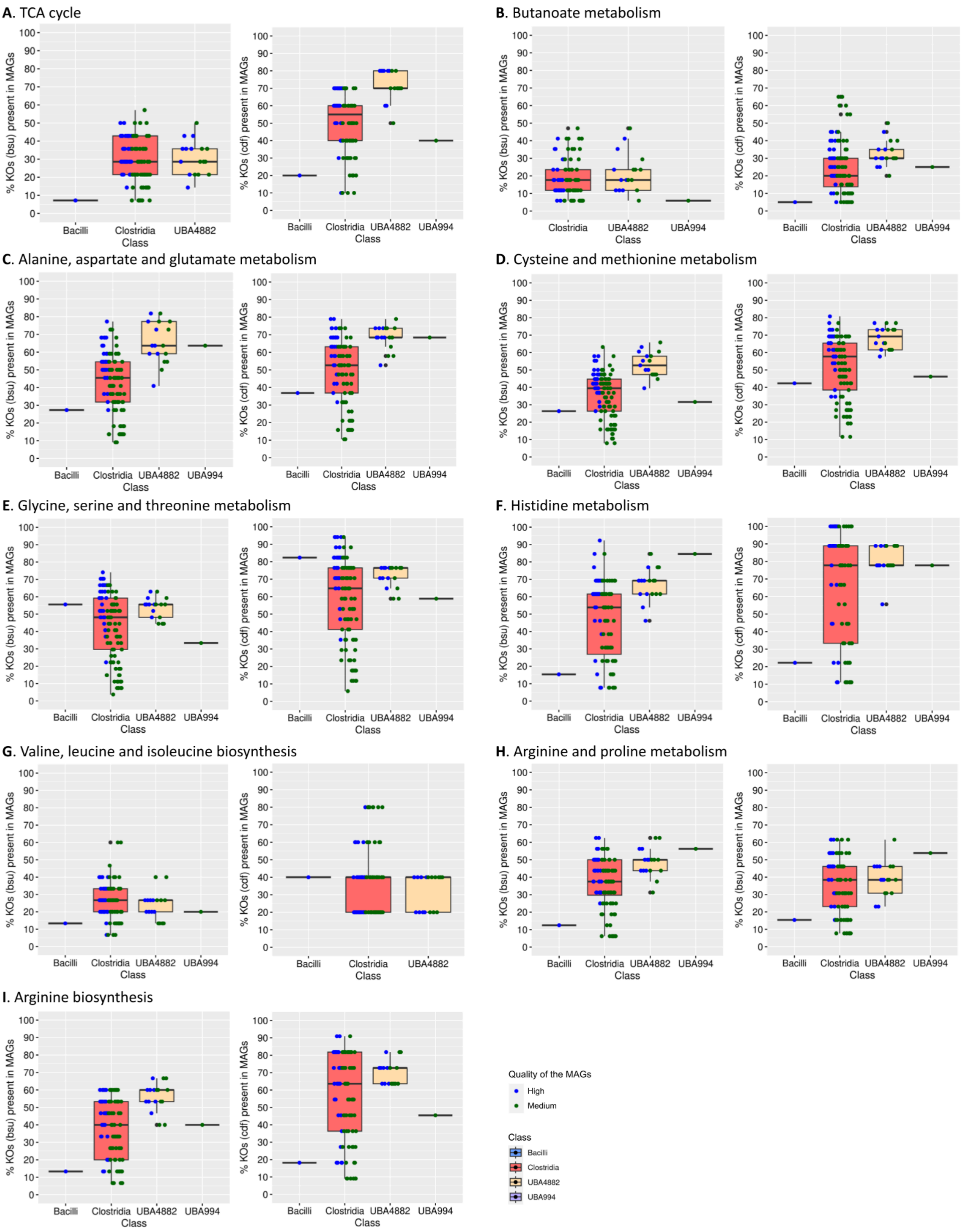

Supplementary Figure 14. Proportion of KOs related to carbohydrate and amino acid pathways in the 124 MAGs not refined taxonomically at the family level. A and B are carbohydrate metabolism pathways, and C-I are amino acid metabolism pathways.
